# Treatment with Tang-luo-ning altered the microRNA expression profile in rats with diabetic peripheral neuropathy

**DOI:** 10.1080/21655979.2020.1797282

**Published:** 2020-07-27

**Authors:** Yixiao Li, Yanbin Gao, Yanbing Gong, Yuxin Guo, Liying Wang, Qin Liu, Feng Chen, Taojing Zhang

**Affiliations:** a Department of Endocrinology, Dongfang Hospital, Beijing University of Chinese Medicine, Beijing, China; b School of Traditional Chinese Medicine, Capital Medical University, Beijing, China

**Keywords:** Peripheral neuropathy, Tang-luo-ning, MicroRNA, microarray, Wnt signaling pathway, neurotrophin

## Abstract

Tang-luo-ning (TLN) is a traditional Chinese herbal recipe that has been used to treat diabetic peripheral neuropathy (DPN); nevertheless, the underlying mechanism remains unclear. This study was aimed to investigate the microRNA (miRNA) expression profile in diabetic rats treated with TLN, and the target genes were predicted. Male Sprague-Dawley rats were randomly divided into control, diabetes, and TLN-treated diabetes groups. Diabetes was induced with streptozotocin, and TLN (5 g/kg/day) was orally given for eight weeks. Then, the sciatic nerves were harvested for miRNA microarray analyses. The differentially expressed miRNAs and their target genes were analyzed. Compared with the control rats, 24 miRNAs were significantly upregulated, and 59 were downregulated in the sciatic nerves of the diabetic rats by more than two folds (all *P* < 0.05). In TLN-treated diabetes rats, 26 miRNAs were upregulated, and 14 were downregulated compared with diabetic rats without TLN treatment (all *P* < 0.05). DPN-induced alterations of the miRNA profile were reversed by the TLN treatment. A total of 1402 target genes were screened. In GO analysis, genes in localization, cytoplasm, and protein binding processes were enriched, and the most significantly enriched pathways included the neurotrophin, Fc epsilon RI, and Wnt signaling pathways. Further analyses revealed that *DVL1* and *NTF3* genes were involved in these pathways. Our findings indicate that TLN may affect the Wnt and neurotrophin pathways by acting on *DVL1* and *NTF3* genes.

## GRAPHICAL ABSTRACT

**Figure:**
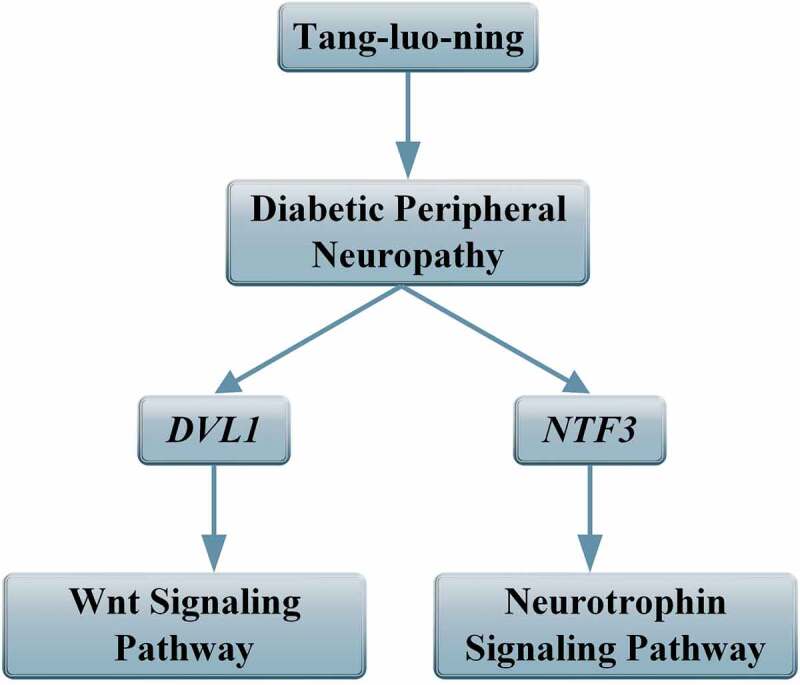

## 1. Introduction

Diabetic peripheral neuropathy (DPN) is a common chronic complication of diabetes and potentially affects the distal sensory, motor, and autonomic nerves. Clinically, DPN is characterized by pain, numbness, and dysfunction of the autonomic nervous system, which seriously impairs individual quality of life [1]. Tang-luo-ning (TLN) is a traditional Chinese herbal recipe that is comprised of *Astragalus root, Fructuscorni*, and *Salvia miltiorrhiza*. In our previous study, the clinical effective rate of TLN for treating DPN was 89.9%, and TLN was significantly superior to mecobalamin [2]. TLN could effectively improve DPN-related symptoms, including numbness, pain, muscle spasms, and impaired tendon reflexes [3]. In animal experiments, TLN could reduce the sorbitol levels in the sciatic nerves of diabetic rats [4]. Additionally, TLN increased the rat serum level of nerve growth factor (NGF) as well as the mRNA expression of NGF in the sciatic nerves [5]. Moreover, TLN also increased the serum insulin-like growth factor-1 (IGF-1) levels and the mRNA expression of IGF-1 in the liver of diabetic rats [6,7]. Further experiments revealed that TLN could activate the Nrg1/ErbB2 and Erk/bad signal pathways to promote Schwann cell survival [8]. In our previous studies, we found that TLN was effective in treating DNP in both clinical and animal experiments [2,4–7]. These conclusions were supported by the experiments conducted by Yang et al. [9]. However, the mechanism underlying the therapeutic effects of TLN in DPN has not yet been clarified.

MicroRNAs (miRNAs) are a type of non-coding RNA of 21–25 nucleotides in length, which are involved in the post-transcriptional regulation of gene expression. miRNAs have been reported to mediate the therapeutic effects of natural herbs [10,11]. In the previous study, we performed a microarray bioinformatics analysis of genes that were differentially regulated by TLN, and cell survival genes (*NRG1, MTOR, GAB1*, and *PI3KCB*) identified from the microarray analysis were validated by qPCR. We found that TLN significantly upregulated the expression levels of *NRG1, MTOR*, and *GAB1* [8].

The aim of this study was to investigate the microRNA (miRNA) expression profile in diabetic rats treated with TLN, and the target genes were predicted.

## 2. Materials and methods

### 2.1. Preparation of TLN

TLN recipe primarily contains ingredients from eight well-known traditional Chinese herbs: *Astragali* (Huangqi), *Rehmanniae* (Dihuang), *Angelicae Sinensis* (Danggui), *Rhizoma Cibotii* (Gouji), *Achyranthis Bidentatae* (Niuxi), *Fructus chaenomelis* (Mugua), *Dipsaci* (Xuduan), and *Rhizoma Salviae* (Danshen). The ratio of the above eight ingredients was 2:2:1:1:1:1:1:1. All herbs were purchased from the Dongfang Hospital of Beijing University of Chinese Medicine (Beijing, China), and all herbs were decocted at a concentration of 10 g/mL (crude drug).

### 2.2. Diabetic rat model and TLN treatment

Male Sprague-Dawley (SD) rats (200–250 g) were purchased from the Vital River Laboratories (Beijing, China). The rats were housed in the Animal Center at the Beijing University of Chinese Medicine, according to the National Standards (Gb14925-2001) [9]. The ambient environment was maintained at a humidity of 60 ± 10% and a temperature of 25 ± 1 °C. Ten rats were randomly chosen as the normal group, and the remaining 20 rats were subjected to the DPN model. The DPN model was induced by intraperitoneal injection of 60 mg/kg streptozotocin (STZ) (Sigma-Aldrich; St. Louis, MO, USA). After 72 hours of injection, the blood glucose was measured; a value of >16.7 mmol/L was defined as diabetes. A total of 20 diabetic rats were randomly divided into groups with (10 rats) or without (10 rats) TLN treatment. TLN was given by gavage at a dose of 5 g/kg/d for eight consecutive weeks. The dosage of TLN was determined based on our experience in previous studies. An equivalent volume of distilled water was used as control. After the last administration, all rats were fasted for 12 hours. Then, all rats were anesthetized using xylazine (10 mg/kg)/ketamine (75 mg/kg), and sciatic nerves were harvested. This study was approved by the Ethics Committee of Beijing University of Chinese Medicine (No. BUCM201738). These animal experiments were conducted according to the NIH guidelines for the care and use of laboratory animals [8].

### 2.3. RNA extraction and miRNA array

Total RNAs were extracted from rat sciatic nerves and quantified by NanoDrop (ND-2100, Thermo Fisher Scientific, Waltham, MA, USA). RNA integrity was assessed using Agilent 2100 (Agilent Technologies, Santa Clara, CA, USA). The sample labeling, microarray hybridization, and washing were performed based on the manufacturer’s standard protocols. Briefly, total RNAs were tailed with Poly A and then labeled with Biotin. The labeled RNAs were then hybridized onto the microarray (Affymetrix miRNA 4.0, Santa Clara, CA, USA). After washing and staining the slides, the arrays were scanned by the Affymetrix Scanner 3000 (Affymetrix, Santa Clara, CA, USA).

### 2.4. Data analysis

Affymetrix GeneChip Command Console software (Version 4.0, Affymetrix, Santa Clara, CA, USA) was used to analyze the array images to obtain raw data. Next, R software was used for the following data analysis. Differentially expressed miRNAs were determined by fold changes as well as *P* values, which was calculated using the *t*-test. A *P* value < 0.05 was considered to indicate statistical significance. Target genes of differentially expressed miRNAs were predicted using three databases (miRanda, miRDB, miRTarbase). GO analysis and KEGG analysis were performed to predict the functions of target genes. Hierarchical clustering was performed to show the distinguishable miRNAs expression patterns among the samples.

## 3. Results

### 3.1. Alterations of the miRNA expression profile after TLN treatment

**Figure 1.**
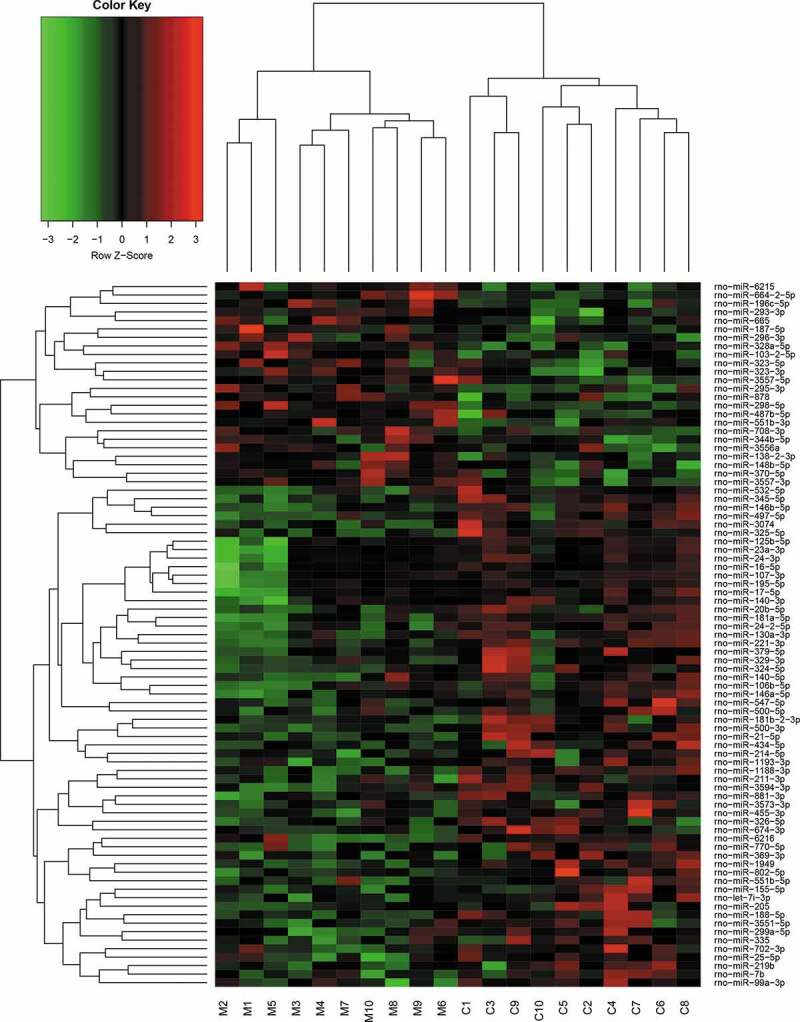
Cluster analysis of miRNAs in the sciatic nerves between control and diabetes rats. Cluster analysis of the 83 differentially expressed genes in the samples of rats with or without STZ-induced diabetes. These 83 genes were clearly clustered into two categories.

**Figure 2.**
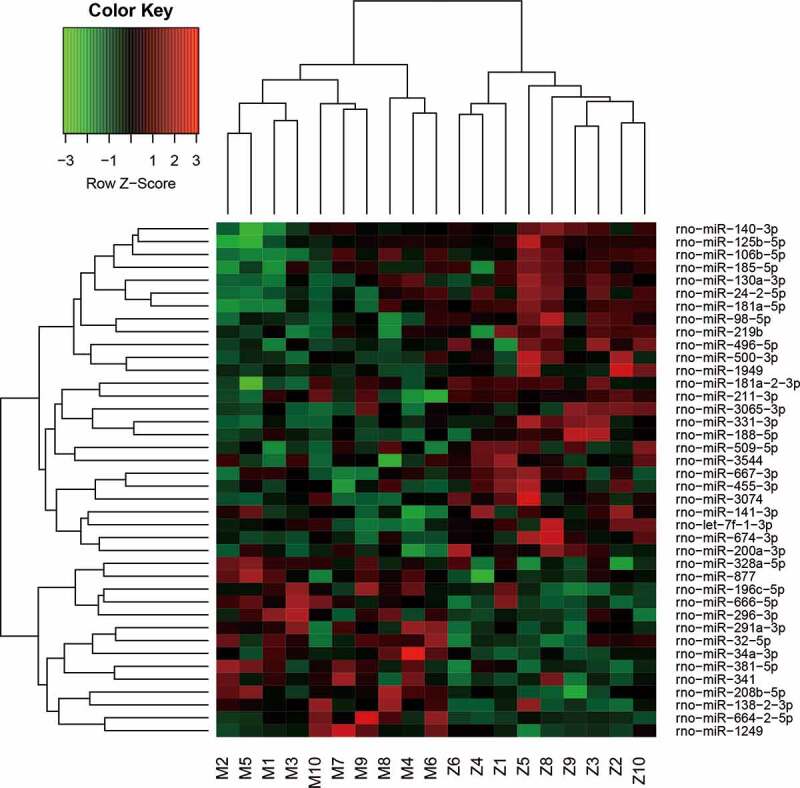
Cluster analysis of miRNAs in the sciatic nerves between diabetic rats treated with or without TLN. Cluster analysis of 40 differentially expressed genes in samples from diabetes rats with or without the TLN treatment.

Compared with the miRNA expression profile in the sciatic nerves of the control rats, 24 miRNAs were significantly upregulated, and 59 miRNAs were downregulated in diabetes rats by more than two folds (all *P* < 0.05, Table 1). Clustering analysis shows that these 83 genes could be clearly clustered into two categories (Figure 1). In diabetes rats treated with TLN, 26 miRNAs were upregulated, and 14 were downregulated compared with diabetic rats without TLN treatment (all *P* < 0.05, Table 2). These 40 genes were also clustered into two categories (Figure 2). Among the differentially expressed genes in the above two analyses, 19 overlapped genes were observed (Table 3). In these 19 genes, the upregulation and downregulation in diabetic rats compared with control rats were reversed by the TLN treatment (Table 3).

**Table 1.** Differential analysis of miRNA expression profiles between control and diabetic rats. Compared with the miRNA expression profile in the sciatic nerves of the control rats, 24 miRNAs were significantly upregulated, and 59 miRNAs were downregulated in diabetic rats by more than 2-fold (all *P* < 0.05).

Transcript ID	*P* value	Regulation	Transcript ID	*P* value	Regulation
rno-miR-298-5p	0.000601	Up	rno-miR-881-3p	0.009382	down
rno-miR-328a-5p	0.001319	Up	rno-miR-140-3p	0.009599	down
rno-miR-6215	0.004605	Up	rno-miR-324-5p	0.01087	down
rno-miR-664-2-5p	0.005968	Up	rno-miR-188-5p	0.012841	down
rno-miR-293-3p	0.007902	Up	rno-miR-20b-5p	0.013061	down
rno-miR-196 c-5p	0.010067	Up	rno-miR-99a-3p	0.013804	down
rno-miR-323-3p	0.012872	Up	rno-miR-329-3p	0.015059	down
rno-miR-3557-3p	0.019802	Up	rno-miR-802-5p	0.015801	down
rno-miR-295-3p	0.027855	Up	rno-miR-125b-5p	0.020173	down
rno-miR-323-5p	0.02912	Up	rno-miR-379-5p	0.02289	down
rno-miR-103-2-5p	0.029818	Up	rno-miR-547-5p	0.023282	down
rno-miR-665	0.030238	Up	rno-miR-195-5p	0.026341	down
rno-miR-148b-5p	0.030289	Up	rno-miR-219b	0.027348	down
rno-miR-708-3p	0.031094	Up	rno-miR-702-3p	0.027864	down
rno-miR-3557-5p	0.03323	Up	rno-miR-434-5p	0.028508	down
rno-miR-370-5p	0.033303	Up	rno-miR-3551-5p	0.028989	down
rno-miR-187-5p	0.033621	Up	rno-miR-211-3p	0.029253	down
rno-miR-878	0.033916	Up	rno-miR-17-5p	0.030546	down
rno-miR-296-3p	0.03675	Up	rno-miR-7b	0.030669	down
rno-miR-138-2-3p	0.038471	Up	rno-miR-181b-2-3p	0.031259	down
rno-miR-487b-5p	0.03894	Up	rno-miR-532-5p	0.032045	down
rno-miR-344b-5p	0.040336	Up	rno-miR-500-5p	0.033759	down
rno-miR-551b-3p	0.043027	Up	rno-miR-335	0.034392	down
rno-miR-3556a	0.043677	Up	rno-miR-3594-3p	0.034587	down
rno-miR-146b-5p	8.56E-05	down	rno-miR-24-3p	0.035025	down
rno-miR-1949	0.000353	down	rno-miR-326-5p	0.035273	down
rno-miR-1188-3p	0.00039	down	rno-miR-16-5p	0.035298	down
rno-miR-500-3p	0.000468	down	rno-miR-369-3p	0.037573	down
rno-miR-221-3p	0.000597	down	rno-miR-23a-3p	0.038335	down
rno-miR-345-5p	0.000873	down	rno-miR-770-5p	0.039743	down
rno-miR-24-2-5p	0.001072	down	rno-miR-214-5p	0.040468	down
rno-miR-181a-5p	0.001144	down	rno-miR-106b-5p	0.040546	down
rno-miR-455-3p	0.001199	down	rno-miR-551b-5p	0.040929	down
rno-miR-497-5p	0.0023	down	rno-miR-674-3p	0.042786	down
rno-miR-6216	0.003279	down	rno-miR-299a-5p	0.043477	down
rno-miR-21-5p	0.00401	down	rno-miR-205	0.043608	down
rno-miR-130a-3p	0.004555	down	rno-miR-155-5p	0.045872	down
rno-miR-3074	0.004559	down	rno-miR-1193-3p	0.046783	down
rno-miR-325-5p	0.004841	down	rno-miR-3573-3p	0.047259	down
rno-let-7i-3p	0.005469	down	rno-miR-25-5p	0.047577	down
rno-miR-146a-5p	0.00609	down	rno-miR-107-3p	0.048387	down
rno-miR-140-5p	0.049036	down

**Table 2.** Differential analysis of miRNA expression profiles between diabetic rats treated with or without TLN. In diabetic rats treated with TLN, 26 miRNAs were upregulated and 14 were downregulated compared with diabetic rats without TLN treatment.

Transcript ID	*P* value	Regulation	Transcript ID	*P* value	Regulation
rno-miR-98-5p	0.001904	up	rno-miR-455-3p	0.042823	up
rno-miR-181a-5p	0.002069	up	rno-miR-1949	0.045168	up
rno-miR-331-3p	0.005843	up	rno-miR-141-3p	0.046962	up
rno-miR-140-3p	0.007649	up	rno-miR-106b-5p	0.047126	up
rno-miR-200a-3p	0.010163	up	rno-miR-185-5p	0.048892	up
rno-miR-674-3p	0.010752	up	rno-miR-3544	0.049457	up
rno-let-7 f-1-3p	0.012635	up	rno-miR-208b-5p	0.002952	down
rno-miR-24-2-5p	0.013539	up	rno-miR-381-5p	0.004767	down
rno-miR-125b-5p	0.013788	up	rno-miR-666-5p	0.006992	down
rno-miR-181a-2-3p	0.016922	up	rno-miR-664-2-5p	0.014131	down
rno-miR-496-5p	0.019889	up	rno-miR-291a-3p	0.014431	down
rno-miR-130a-3p	0.021459	up	rno-miR-196 c-5p	0.016558	down
rno-miR-3074	0.021846	up	rno-miR-34a-3p	0.020563	down
rno-miR-3065-3p	0.022887	up	rno-miR-296-3p	0.021954	down
rno-miR-211-3p	0.027288	up	rno-miR-328a-5p	0.023016	down
rno-miR-509-5p	0.032447	up	rno-miR-138-2-3p	0.024929	down
rno-miR-219b	0.035383	up	rno-miR-877	0.029105	down
rno-miR-667-3p	0.035579	up	rno-miR-341	0.040315	down
rno-miR-188-5p	0.036619	up	rno-miR-32-5p	0.042423	down
rno-miR-500-3p	0.03719	up	rno-miR-1249	0.042709	down

**Table 3.** The common miRNAs in both differential analyses. In these 19 genes, the upregulation and downregulation in diabetic rats compared with control rats were reversed by treatment with TLN.

y	Regulation (diabetic rats *vs*. control)	Regulation (diabetic rats treated with or without TLN)
rno-miR-106b-5p	down	Up
rno-miR-125b-5p	down	Up
rno-miR-130a-3p	down	Up
rno-miR-138-2-3p	Up	Down
rno-miR-140-3p	down	Up
rno-miR-181a-5p	down	Up
rno-miR-188-5p	down	Up
rno-miR-1949	down	Up
rno-miR-196 c-5p	Up	Down
rno-miR-211-3p	down	Up
rno-miR-219b	down	Up
rno-miR-24-2-5p	down	Up
rno-miR-296-3p	Up	Down
rno-miR-3074	down	Up
rno-miR-328a-5p	Up	Down
rno-miR-455-3p	down	Up
rno-miR-500-3p	down	Up
rno-miR-664-2-5p	Up	Down
rno-miR-674-3p	down	Up

### 3.2. Prediction of target genes

Target genes of the differentially expressed miRNAs were predicted using the miRanda, miRDB, and miRTarbase databases, with at least two databases included as the screening criteria. A total of 1402 target genes were screened (Table S1).

**Figure 3.**
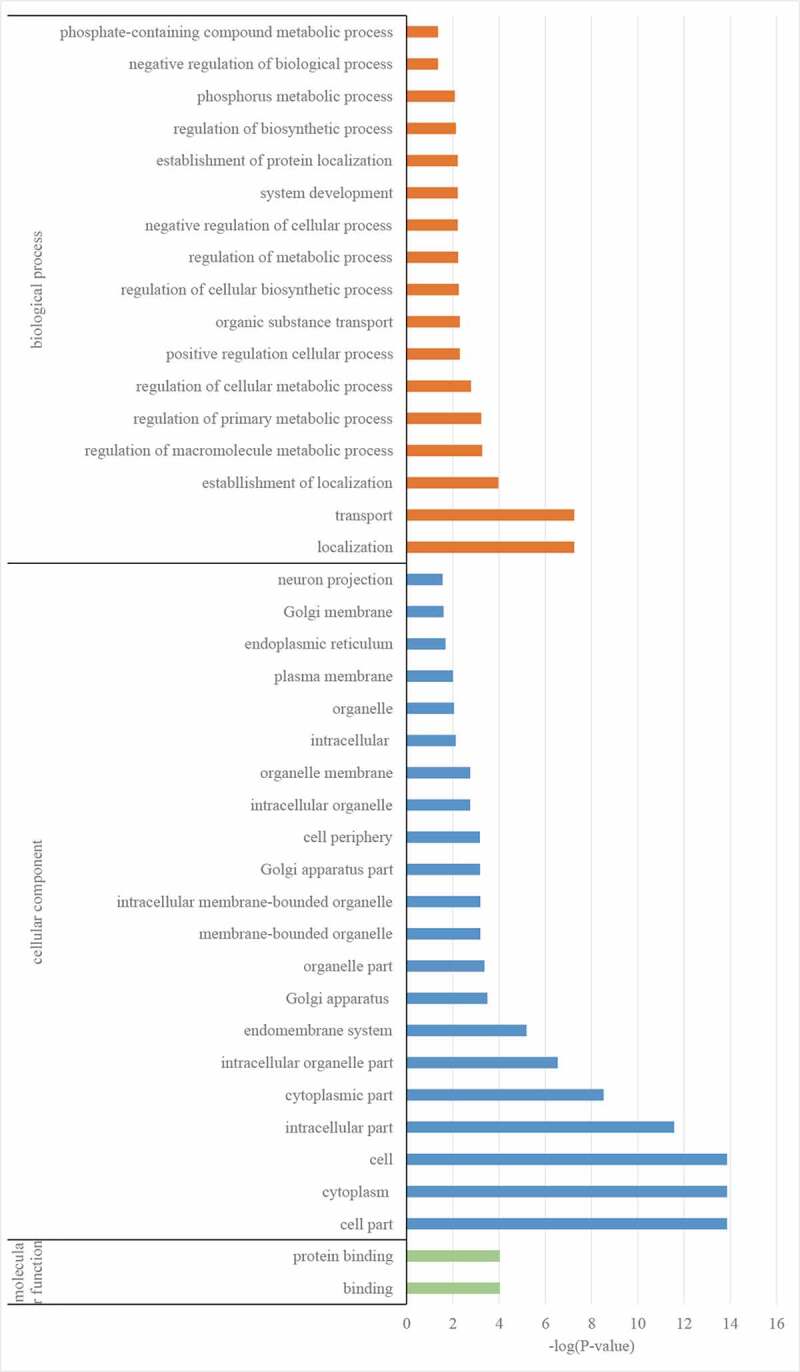
GO analysis of miRNA-targeted genes between diabetes rats treated with or without TLN. Orange bars represent biological processes, blue bars represent cellular components, and green bars represent molecular functions.

GO analysis of the target genes was performed, which were designated to the categories of biological process, cellular component, and molecular function. In the biological process category, the most significantly changed items between diabetes rats treated with or without TLN were localization, transport, and establishment of localization (Figure 3). In the cellular component category, the most significantly altered items were cell part, cytoplasm, and cell (Figure 3). The most significant entries in the molecular function were binding and protein binding (Figure 3).

We performed a pathway analysis of the miRNA-targeted genes using the KEGG database. The number of target genes in each pathway was counted, and the significance of the enrichment of the target genes was analyzed. The top 10 significantly enriched pathways were presented in Table 4. After the TLN treatment, the most significantly enriched pathways were metabolic pathway, endocytosis, and cytokine-cytokine receptor interaction (Table 4).

**Table 4.** KEGG pathway analysis of target genes between the TLN-treated diabetes group and vehicle-treated diabetes group.

Pathway Name	#Gene	Entrez Gene	Adjusted *P* value
Metabolic pathways	77	300886 64304 25428 289827 64203 5065525095 316067 24311 500826 83505 29505129592 64442 406165 299857 64189 25288310848 296851 29632 54398 266685191575 298490 81919 81925 310378298934 171142 313878 308589 246232156275 81519 292728 25044 24188 114860116676 65196 29194 24513 29254 30541925283 300741 64043 298410 83626 35322924184 117596 81924 24334 305889 17108229582 116664 24479 314641 29637 30392365183 360426 299201 24465 298098 29758362720 64134 64191 84029 113976 2510483627 306012	< 0.0001
Endocytosis	22	140694 360834 25645 140670 54241 29591290775 282834 246310 361916 29717381809 404781 81830 25751 60443 81516192204 24176 54190 25718 314641	< 0.0001
Cytokine-cytokine receptor interaction	15	29201 360750 29591 366518 25341 2483525647 252963 24499 24772 360457 2538589814 81809 245920	0.0001
Gastric acid secretion	9	64508 24681 84396 24242 65272 2526225650 501563 24213	0.0001
Bile secretion	9	25428 64508 25293 84396 25650 50156324213 25073 83500	0.0001
Neurotrophin signaling pathway	17	362418 116551 24242 25050 25054 24516297173 170851 295347 50658 24605 81737170820 29513 170538 56010 25385	< 0.0001
Chagas disease (American trypanosomiasis)	10	24672 50658 117104 29591 24835 2951324516 64044 25385 81809	0.0002
Fc epsilon RI signaling pathway	12	24605 50658 360426 116551 24681 2483529513 170538 81515 25155 29340 170851	< 0.0001
Pancreatic cancer	8	50658 29591 84014 25125 360457 24827170851 81809	0.0003
Wnt signaling pathway	12	24672 50658 64152 29748 24681 65137501563 24516 361765 83721 297173140941	0.0003

### 3.3. Intersection analysis of GO analysis and pathway analysis

**Figure 4.**
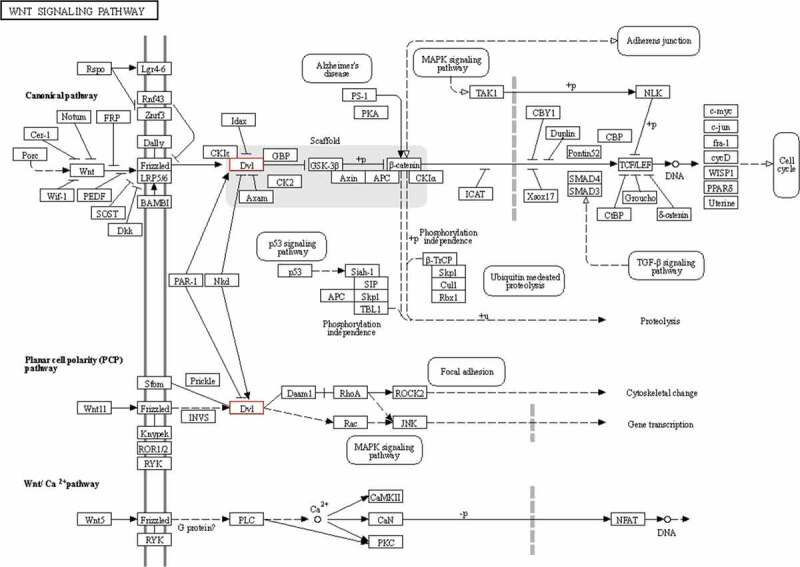
*DVL1* in the Wnt signaling pathway. Schematic diagram of the role of *DVL1* in the Wnt signaling pathway.

**Figure 5.**
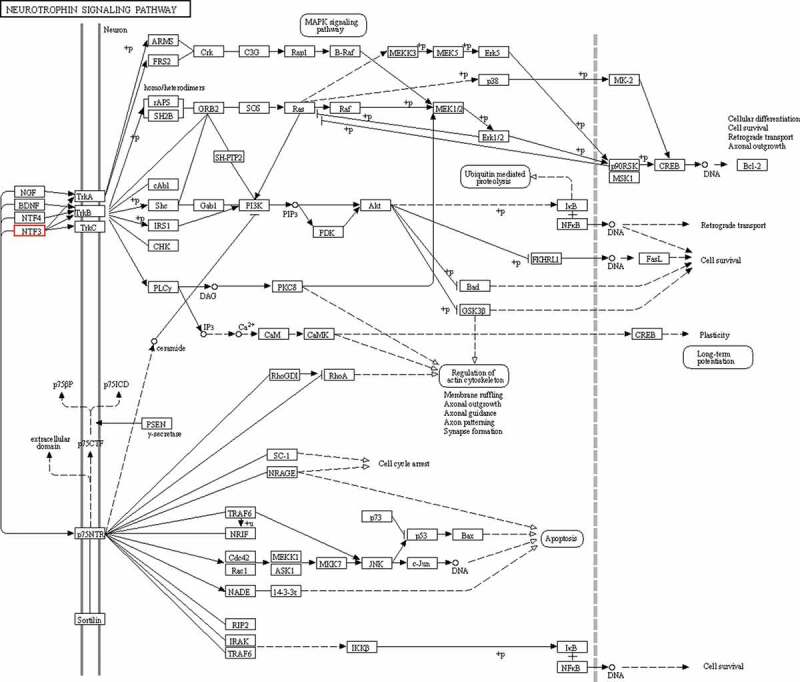
*NTF3* in the neurotrophin signaling pathway. Schematic diagram of the role of *NTF3* in the neurotrophin signaling pathway.

We performed an intersection analysis of GO and pathway analyses. Between the normal and diabetes groups, there were 469 intersection genes. Between the diabetic rats treated with or without TLN, there were 235 intersection genes. There were 124 overlapped intersection genes between the above two analyses. We further analyzed the top 10 pathways and identified two genes, *DVL1* and *NTF3*, which were involved in the Wnt (Figure 4) and neurotrophin (Figure 5) signaling pathways.

## 4. Discussion

DPN is a chronic microvascular complication of diabetes. According to global epidemiological surveys, at least 50% of patients with type 1 or type 2 diabetes suffer from DPN, which is one of the major causes of amputation in diabetic patients [12]. Clinical manifestations in patients with DPN include decreased motor and sensory nerve conduction velocity, microvascular and microcirculatory dysfunction, the increased threshold of vibration and thermal pain perception, progressive sensory loss, and peripheral nerve degeneration. Most patients with DPN usually have self-described symptoms such as paresthesia, allodynia, hyperalgesia, and spontaneous pain [13–18].

In the differential expression analysis of the miRNA expression profile, we found that 19 miRNAs were overlapped in the comparisons of control *vs*. diabetic rats and diabetic rats treated with vehicle *vs*. diabetic rats treated with TLN. In addition, diabetes-induced changes of these 19 miRNAs were reversed by the TLN treatment, suggesting that the therapeutic effects of TLN on DPN may be associated with an altered miRNA expression profile. In the GO analysis of miRNA target genes, there was a significant gene enrichment in the localization, cytoplasm, and protein binding processes, suggesting that TLN may improve the symptoms of DPN by affecting these three cell processes. In the pathway analysis of miRNA-targeted genes in diabetes rats treated with *vs*. without TLN, the neurotrophin signaling pathway, Fc epsilon RI signaling pathway, and Wnt signaling pathway are most significantly enriched.

Through further pathway and gene analysis, *DVL1* from the Wnt signaling pathway and neurotrophin-3 (*NTF3*) from the neurotrophin signaling pathway were identified. A previous study found that the Wnt signaling pathway may mediate oxidized low-density lipoprotein (ox-LDL)-induced endothelial cell damage through oxidative stress and that endothelial cell damage can be ameliorated by suppressing oxidative stress and inhibiting the Wnt signaling pathway [19,20]. The protein encoded by the *DVL1* gene is a cytoplasmic phosphoprotein that plays an important role in the Wnt signaling pathway [20]. Figure 4 shows the relationship between *DVL1* and other components in the Wnt pathway. These results suggest that treatment with TLN may influence the *DVL1* expression and then affect the Wnt signaling pathway, resulting in reduced oxidative stress and improved symptoms of DPN. According to previous studies, *NTF3* belongs to the nerve growth factor family and is widely expressed in the central nervous system and surrounding tissues [21]. *NTF3* can induce the growth of neurites and promote the expression of key enzymes involved in neurotransmitter synthesis. *NTF3* plays an important role in the development, differentiation, growth, regeneration, and functional characteristics of the central and peripheral neurons [22–25]. A lack of *NTF3* is associated with a variety of neurological disorders [25–28]. In addition, *NTF3* plays a critical role in the survival and function of sensory neurons and sympathetic neurons. Studies have also shown that *NTF3* has therapeutic effects on peripheral neuropathy [29–31]. Notably, *NTF3* protects sensory neurons in diabetes rats and promotes axonal regeneration in diabetic lesions [32,33]. Taken together, *NTF3* may mediate the therapeutic effects of TLN on DPN. In this study, we predicted the target genes but did not quantify the protein expression levels. In the future, we will conduct validation and functional experiments for specific genes.

## 5. Conclusion

Our findings indicate that TLN may affect the Wnt and neurotrophin pathways by acting on *DVL1* and *NTF3* genes. Moreover, TLN may improve the symptoms of DPN by affecting the localization, cytoplasm, and protein binding processes.

## Supplementary Material

Supplemental Material

## Data Availability

All data generated or analyzed during this study are included in this manuscript.

## Research highlights

1. Tang-luo-ning (TLN) has been used to treat diabetic peripheral neuropathy (DPN);
2. The underlying mechanism of TLN treating DPN remains unclear;
3. We investigated the microRNA (miRNA) expression profile using microarrays;
4. TLN may affect the Wnt and neurotrophin pathways by acting on *DVL1* and *NTF3* genes.
