# Supplementary material for: Treatment with Tang-luo-ning altered the microRNA expression profile in rats with diabetic peripheral neuropathy
Source: Bioengineered. 2020 Jul 27;11(1):841–51. doi: 10.1080/21655979.2020.1797282 (PMC8291862; doi:10.1080/21655979.2020.1797282)
Supplement: Supplemental Material [file KBIE_A_1797282_SM7256.docx]

| **Supplemental Table 1.** Target genes of identified miRNA | | | |
| --- | --- | --- | --- |
| Transcript ID | Accession number | Gene_ID | Gene_Symbol |
| MIMAT0000811 | rno-miR-32-5p | 361930 | Bbs7 |
| MIMAT0000811 | rno-miR-32-5p | 362320 | Fam133b |
| MIMAT0000811 | rno-miR-32-5p | 29642 | Slc38a2 |
| MIMAT0000811 | rno-miR-32-5p | 290364 | Itm2b |
| MIMAT0000811 | rno-miR-32-5p | 246060 | Cdkn1c |
| MIMAT0000811 | rno-miR-32-5p | 312694 | Necap1 |
| MIMAT0000811 | rno-miR-32-5p | 25247 | Nsg1 |
| MIMAT0000811 | rno-miR-32-5p | 29619 | Btg2 |
| MIMAT0000811 | rno-miR-32-5p | 300741 | Mpi |
| MIMAT0000811 | rno-miR-32-5p | 314641 | Pip5k1c |
| MIMAT0000811 | rno-miR-32-5p | 289827 | Ugp2 |
| MIMAT0000811 | rno-miR-32-5p | 307288 | Gramd3 |
| MIMAT0000811 | rno-miR-32-5p | 362235 | Tmem90b |
| MIMAT0000811 | rno-miR-32-5p | 360854 | Arpc5 |
| MIMAT0000811 | rno-miR-32-5p | 362339 | Creb3l2 |
| MIMAT0000811 | rno-miR-32-5p | 363448 | Dynlt3 |
| MIMAT0000811 | rno-miR-32-5p | 29362 | Tef |
| MIMAT0000811 | rno-miR-32-5p | 24311 | Ddc |
| MIMAT0000811 | rno-miR-32-5p | 303059 | Ccnjl |
| MIMAT0000811 | rno-miR-32-5p | 117536 | Nelf |
| MIMAT0000811 | rno-miR-32-5p | 171373 | Slc12a5 |
| MIMAT0000811 | rno-miR-32-5p | 63840 | Per2 |
| MIMAT0000811 | rno-miR-32-5p | 29140 | Snn |
| MIMAT0000811 | rno-miR-32-5p | 24477 | Ibsp |
| MIMAT0000811 | rno-miR-32-5p | 288772 | Spryd4 |
| MIMAT0000811 | rno-miR-32-5p | 25385 | Faslg |
| MIMAT0000811 | rno-miR-32-5p | 171109 | Dusp5 |
| MIMAT0000811 | rno-miR-32-5p | 314949 | Rad21 |
| MIMAT0000811 | rno-miR-32-5p | 309446 | Hps6 |
| MIMAT0000811 | rno-miR-32-5p | 29628 | Gria3 |
| MIMAT0000811 | rno-miR-32-5p | 300463 | Herpud2 |
| MIMAT0000811 | rno-miR-32-5p | 83612 | Slc32a1 |
| MIMAT0000811 | rno-miR-32-5p | 24908 | Dnajb9 |
| MIMAT0000811 | rno-miR-32-5p | 84029 | Hao2 |
| MIMAT0000811 | rno-miR-32-5p | 25181 | Bgn |
| MIMAT0000811 | rno-miR-32-5p | 282835 | Wrnip1 |
| MIMAT0000811 | rno-miR-32-5p | 25159 | Gata2 |
| MIMAT0000811 | rno-miR-32-5p | 362535 | RGD1308059 |
| MIMAT0000811 | rno-miR-32-5p | 29187 | Cd69 |
| MIMAT0000811 | rno-miR-32-5p | 306439 | Gpm6a |
| MIMAT0000811 | rno-miR-32-5p | 116500 | Snap29 |
| MIMAT0000811 | rno-miR-32-5p | 81924 | Cyp8b1 |
| MIMAT0000811 | rno-miR-32-5p | 24948 | Fmr1 |
| MIMAT0000811 | rno-miR-32-5p | 59112 | Hand1 |
| MIMAT0000811 | rno-miR-32-5p | 192126 | Dab2ip |
| MIMAT0000811 | rno-miR-32-5p | 299261 | Moap1 |
| MIMAT0000811 | rno-miR-32-5p | 25521 | Prkar1b |
| MIMAT0000811 | rno-miR-32-5p | 286932 | Vps54 |
| MIMAT0000811 | rno-miR-32-5p | 171548 | Dkk3 |
| MIMAT0000811 | rno-miR-32-5p | 306165 | Gpr180 |
| MIMAT0000811 | rno-miR-32-5p | 294978 | RGD1307100 |
| MIMAT0000811 | rno-miR-32-5p | 362699 | Yipf4 |
| MIMAT0000811 | rno-miR-32-5p | 50677 | Ptpro |
| MIMAT0000811 | rno-miR-32-5p | 501668 | Dmrtc1a |
| MIMAT0000811 | rno-miR-32-5p | 252856 | Tcf21 |
| MIMAT0000811 | rno-miR-32-5p | 64508 | Adcy3 |
| MIMAT0000811 | rno-miR-32-5p | 314992 | RGD1310852 |
| MIMAT0000811 | rno-miR-32-5p | 290985 | Isca1 |
| MIMAT0000811 | rno-miR-32-5p | 499108 | Sertad3 |
| MIMAT0000811 | rno-miR-32-5p | 64194 | Insig1 |
| MIMAT0000811 | rno-miR-32-5p | 298490 | Ppcs |
| MIMAT0000811 | rno-miR-32-5p | 54348 | Stk39 |
| MIMAT0000811 | rno-miR-32-5p | 25615 | Sdc2 |
| MIMAT0000811 | rno-miR-32-5p | 60331 | Atxn3 |
| MIMAT0000811 | rno-miR-32-5p | 170842 | Tob1 |
| MIMAT0000811 | rno-miR-32-5p | 171101 | Grp |
| MIMAT0000811 | rno-miR-32-5p | 619566 | Cdca7l |
| MIMAT0000811 | rno-miR-32-5p | 316742 | Tgif1 |
| MIMAT0000811 | rno-miR-32-5p | 85431 | Nox4 |
| MIMAT0000811 | rno-miR-32-5p | 114505 | Klf4 |
| MIMAT0000811 | rno-miR-32-5p | 309145 | RGD1311946 |
| MIMAT0000811 | rno-miR-32-5p | 171086 | Trak2 |
| MIMAT0000811 | rno-miR-32-5p | 29194 | Chka |
| MIMAT0000811 | rno-miR-32-5p | 315548 | Srpr |
| MIMAT0000811 | rno-miR-32-5p | 24588 | Nefm |
| MIMAT0000811 | rno-miR-32-5p | 84352 | Col1a2 |
| MIMAT0000811 | rno-miR-32-5p | 25428 | Cyp7a1 |
| MIMAT0000811 | rno-miR-32-5p | 84588 | Cldn11 |
| MIMAT0000811 | rno-miR-32-5p | 170946 | Lkap |
| MIMAT0000811 | rno-miR-32-5p | 500069 | Tsga14 |
| MIMAT0000811 | rno-miR-32-5p | 266777 | Nptx1 |
| MIMAT0000811 | rno-miR-32-5p | 170551 | Slc5a6 |
| MIMAT0000811 | rno-miR-32-5p | 58853 | Nr4a3 |
| MIMAT0000811 | rno-miR-32-5p | 297173 | RSA-14-44 |
| MIMAT0000811 | rno-miR-32-5p | 65272 | Kcnk10 |
| MIMAT0000811 | rno-miR-32-5p | 286897 | Dpy30 |
| MIMAT0000811 | rno-miR-32-5p | 25262 | Itpr1 |
| MIMAT0000811 | rno-miR-32-5p | 29254 | Mgll |
| MIMAT0000811 | rno-miR-32-5p | 79428 | Luzp1 |
| MIMAT0000811 | rno-miR-32-5p | 500400 | Fam110b |
| MIMAT0000811 | rno-miR-32-5p | 84007 | C3ar1 |
| MIMAT0000819 | rno-miR-98-5p | 25066 | PVR |
| MIMAT0000819 | rno-miR-98-5p | 363232 | Bzw1 |
| MIMAT0000819 | rno-miR-98-5p | 29524 | Limk2 |
| MIMAT0000819 | rno-miR-98-5p | 497899 | LOC497899 |
| MIMAT0000819 | rno-miR-98-5p | 89868 | Sec16b |
| MIMAT0000819 | rno-miR-98-5p | 297383 | Rtkn |
| MIMAT0000819 | rno-miR-98-5p | 85264 | Abcg1 |
| MIMAT0000819 | rno-miR-98-5p | 29591 | Tgfbr1 |
| MIMAT0000819 | rno-miR-98-5p | 58949 | Ptafr |
| MIMAT0000819 | rno-miR-98-5p | 29140 | Snn |
| MIMAT0000819 | rno-miR-98-5p | 81826 | Slc20a1 |
| MIMAT0000819 | rno-miR-98-5p | 313668 | Ddi2 |
| MIMAT0000819 | rno-miR-98-5p | 114591 | Dpp3 |
| MIMAT0000819 | rno-miR-98-5p | 309082 | Clrn3 |
| MIMAT0000819 | rno-miR-98-5p | 79212 | Slc6a1 |
| MIMAT0000819 | rno-miR-98-5p | 171142 | Ehhadh |
| MIMAT0000819 | rno-miR-98-5p | 288772 | Spryd4 |
| MIMAT0000819 | rno-miR-98-5p | 25385 | Faslg |
| MIMAT0000819 | rno-miR-98-5p | 316583 | B3gnt7 |
| MIMAT0000819 | rno-miR-98-5p | 114514 | Clasp2 |
| MIMAT0000819 | rno-miR-98-5p | 246074 | Scd1 |
| MIMAT0000819 | rno-miR-98-5p | 29683 | Klrc1 |
| MIMAT0000819 | rno-miR-98-5p | 361401 | Pla2g15 |
| MIMAT0000819 | rno-miR-98-5p | 362828 | Sppl2b |
| MIMAT0000819 | rno-miR-98-5p | 298609 | Efhd2 |
| MIMAT0000819 | rno-miR-98-5p | 500909 | Ccdc134 |
| MIMAT0000819 | rno-miR-98-5p | 83823 | Gipc1 |
| MIMAT0000819 | rno-miR-98-5p | 63886 | Abcb9 |
| MIMAT0000819 | rno-miR-98-5p | 266764 | Tbkbp1 |
| MIMAT0000819 | rno-miR-98-5p | 25645 | Adrb3 |
| MIMAT0000819 | rno-miR-98-5p | 619573 | Fam104a |
| MIMAT0000819 | rno-miR-98-5p | 286910 | Nid67 |
| MIMAT0000819 | rno-miR-98-5p | 360646 | Limd2 |
| MIMAT0000819 | rno-miR-98-5p | 116721 | Abcc5 |
| MIMAT0000819 | rno-miR-98-5p | 291948 | Pgrmc1 |
| MIMAT0000819 | rno-miR-98-5p | 64088 | Snx16 |
| MIMAT0000819 | rno-miR-98-5p | 29434 | Rasgrp1 |
| MIMAT0000819 | rno-miR-98-5p | 288774 | Stat2 |
| MIMAT0000819 | rno-miR-98-5p | 58840 | Mapk6 |
| MIMAT0000819 | rno-miR-98-5p | 24176 | Adrb2 |
| MIMAT0000819 | rno-miR-98-5p | 306012 | Polr3d |
| MIMAT0000819 | rno-miR-98-5p | 299602 | Cdc34 |
| MIMAT0000819 | rno-miR-98-5p | 64025 | Cd244 |
| MIMAT0000819 | rno-miR-98-5p | 311331 | Spint1 |
| MIMAT0000819 | rno-miR-98-5p | 361274 | Nudt5 |
| MIMAT0000819 | rno-miR-98-5p | 619549 | Ppapdc2 |
| MIMAT0000819 | rno-miR-98-5p | 295395 | Rtcd1 |
| MIMAT0000819 | rno-miR-98-5p | 25505 | P2rx1 |
| MIMAT0000819 | rno-miR-98-5p | 171152 | Taf9b |
| MIMAT0000819 | rno-miR-98-5p | 315611 | Scn4b |
| MIMAT0000819 | rno-miR-98-5p | 292022 | Ddx19a |
| MIMAT0000819 | rno-miR-98-5p | 315702 | RGD1305464 |
| MIMAT0000819 | rno-miR-98-5p | 361527 | Pld3 |
| MIMAT0000819 | rno-miR-98-5p | 140914 | Olr1 |
| MIMAT0000819 | rno-miR-98-5p | 25113 | Ddn |
| MIMAT0000819 | rno-miR-98-5p | 366518 | Tnfrsf14 |
| MIMAT0000819 | rno-miR-98-5p | 79218 | Gng5 |
| MIMAT0000819 | rno-miR-98-5p | 83626 | Ugcg |
| MIMAT0000819 | rno-miR-98-5p | 498185 | Slc24a6 |
| MIMAT0000819 | rno-miR-98-5p | 313033 | Xkr8 |
| MIMAT0000819 | rno-miR-98-5p | 296616 | Cercam |
| MIMAT0000819 | rno-miR-98-5p | 64553 | Akap6 |
| MIMAT0000819 | rno-miR-98-5p | 117026 | Apbb3 |
| MIMAT0000819 | rno-miR-98-5p | 315760 | Parp16 |
| MIMAT0000819 | rno-miR-98-5p | 364719 | Prss16 |
| MIMAT0000819 | rno-miR-98-5p | 29745 | Sema4f |
| MIMAT0000819 | rno-miR-98-5p | 140934 | Ikbkap |
| MIMAT0000819 | rno-miR-98-5p | 360230 | Stk40 |
| MIMAT0000819 | rno-miR-98-5p | 156435 | Tmprss2 |
| MIMAT0000819 | rno-miR-98-5p | 308995 | Itgal |
| MIMAT0000819 | rno-miR-98-5p | 170551 | Slc5a6 |
| MIMAT0000819 | rno-miR-98-5p | 313782 | Fbxl12 |
| MIMAT0000819 | rno-miR-98-5p | 114860 | Gale |
| MIMAT0000819 | rno-miR-98-5p | 266770 | Soat2 |
| MIMAT0000819 | rno-miR-98-5p | 24605 | Nras |
| MIMAT0000819 | rno-miR-98-5p | 361110 | Tmem110 |
| MIMAT0000819 | rno-miR-98-5p | 317630 | Pldn |
| MIMAT0000819 | rno-miR-98-5p | 85246 | Gas7 |
| MIMAT0000819 | rno-miR-98-5p | 311166 | Clp1 |
| MIMAT0000819 | rno-miR-98-5p | 85262 | Slc25a27 |
| MIMAT0000819 | rno-miR-98-5p | 300095 | Srebf2 |
| MIMAT0000825 | rno-miR-106b-5p | 171581 | Rhov |
| MIMAT0000825 | rno-miR-106b-5p | 310377 | RGD1306227 |
| MIMAT0000825 | rno-miR-106b-5p | 363035 | Zbtb44 |
| MIMAT0000825 | rno-miR-106b-5p | 393092 | Pcdhac2 |
| MIMAT0000825 | rno-miR-106b-5p | 295692 | Nup35 |
| MIMAT0000825 | rno-miR-106b-5p | 308023 | Ssx2ip |
| MIMAT0000825 | rno-miR-106b-5p | 297096 | Snx10 |
| MIMAT0000825 | rno-miR-106b-5p | 24766 | Scn2a1 |
| MIMAT0000825 | rno-miR-106b-5p | 116778 | Pcdha10 |
| MIMAT0000825 | rno-miR-106b-5p | 25044 | Sds |
| MIMAT0000825 | rno-miR-106b-5p | 365493 | RGD1308127 |
| MIMAT0000825 | rno-miR-106b-5p | 25549 | Slc18a2 |
| MIMAT0000825 | rno-miR-106b-5p | 298894 | Mycn |
| MIMAT0000825 | rno-miR-106b-5p | 54702 | Egln3 |
| MIMAT0000825 | rno-miR-106b-5p | 65168 | Scamp2 |
| MIMAT0000825 | rno-miR-106b-5p | 294287 | Phf1 |
| MIMAT0000825 | rno-miR-106b-5p | 171121 | Ppp6c |
| MIMAT0000825 | rno-miR-106b-5p | 362720 | Rrm2 |
| MIMAT0000825 | rno-miR-106b-5p | 314457 | Ankrd9 |
| MIMAT0000825 | rno-miR-106b-5p | 246310 | Arfgap1 |
| MIMAT0000825 | rno-miR-106b-5p | 393086 | Pcdha2 |
| MIMAT0000825 | rno-miR-106b-5p | 297337 | Rnf181 |
| MIMAT0000825 | rno-miR-106b-5p | 83712 | Rbbp7 |
| MIMAT0000825 | rno-miR-106b-5p | 25694 | Has2 |
| MIMAT0000825 | rno-miR-106b-5p | 25050 | Camk4 |
| MIMAT0000825 | rno-miR-106b-5p | 303293 | Cyb5d2 |
| MIMAT0000825 | rno-miR-106b-5p | 293507 | Sept1 |
| MIMAT0000825 | rno-miR-106b-5p | 393085 | Pcdha1 |
| MIMAT0000825 | rno-miR-106b-5p | 116741 | Pcdha4 |
| MIMAT0000825 | rno-miR-106b-5p | 117107 | Zbtb7a |
| MIMAT0000825 | rno-miR-106b-5p | 308937 | Wee1 |
| MIMAT0000825 | rno-miR-106b-5p | 287276 | Sar1b |
| MIMAT0000825 | rno-miR-106b-5p | 246232 | Uxs1 |
| MIMAT0000825 | rno-miR-106b-5p | 297393 | Nagk |
| MIMAT0000825 | rno-miR-106b-5p | 64476 | Mfn2 |
| MIMAT0000825 | rno-miR-106b-5p | 116779 | Pcdha12 |
| MIMAT0000825 | rno-miR-106b-5p | 306809 | Bicd2 |
| MIMAT0000825 | rno-miR-106b-5p | 116780 | Pcdha3 |
| MIMAT0000825 | rno-miR-106b-5p | 311872 | Zbtb43 |
| MIMAT0000825 | rno-miR-106b-5p | 294568 | Wasf1 |
| MIMAT0000825 | rno-miR-106b-5p | 65208 | Dpysl5 |
| MIMAT0000825 | rno-miR-106b-5p | 312135 | Tmem168 |
| MIMAT0000825 | rno-miR-106b-5p | 114591 | Dpp3 |
| MIMAT0000825 | rno-miR-106b-5p | 170904 | Stk17b |
| MIMAT0000825 | rno-miR-106b-5p | 171093 | Shank2 |
| MIMAT0000825 | rno-miR-106b-5p | 113976 | Acsl4 |
| MIMAT0000825 | rno-miR-106b-5p | 24874 | Vhl |
| MIMAT0000825 | rno-miR-106b-5p | 361916 | Chmp4c |
| MIMAT0000825 | rno-miR-106b-5p | 315265 | Twf1 |
| MIMAT0000825 | rno-miR-106b-5p | 362007 | Sike |
| MIMAT0000825 | rno-miR-106b-5p | 314949 | Rad21 |
| MIMAT0000825 | rno-miR-106b-5p | 50594 | Nbl1 |
| MIMAT0000825 | rno-miR-106b-5p | 393088 | Pcdha6 |
| MIMAT0000825 | rno-miR-106b-5p | 303584 | Plekhm1 |
| MIMAT0000825 | rno-miR-106b-5p | 315134 | Josd1 |
| MIMAT0000825 | rno-miR-106b-5p | 295674 | Plekha3 |
| MIMAT0000825 | rno-miR-106b-5p | 364879 | Isoc1 |
| MIMAT0000825 | rno-miR-106b-5p | 58967 | Sfmbt1 |
| MIMAT0000825 | rno-miR-106b-5p | 24908 | Dnajb9 |
| MIMAT0000825 | rno-miR-106b-5p | 305482 | Mtmr3 |
| MIMAT0000825 | rno-miR-106b-5p | 24508 | Irf1 |
| MIMAT0000825 | rno-miR-106b-5p | 65048 | Fat2 |
| MIMAT0000825 | rno-miR-106b-5p | 362129 | Gtdc1 |
| MIMAT0000825 | rno-miR-106b-5p | 85245 | Kpna2 |
| MIMAT0000825 | rno-miR-106b-5p | 64528 | Golga2 |
| MIMAT0000825 | rno-miR-106b-5p | 25485 | Myo1d |
| MIMAT0000825 | rno-miR-106b-5p | 24772 | Cxcl12 |
| MIMAT0000825 | rno-miR-106b-5p | 25139 | Slc2a4 |
| MIMAT0000825 | rno-miR-106b-5p | 25584 | F3 |
| MIMAT0000825 | rno-miR-106b-5p | 362979 | Panx2 |
| MIMAT0000825 | rno-miR-106b-5p | 29187 | Cd69 |
| MIMAT0000825 | rno-miR-106b-5p | 25202 | Gucy1b3 |
| MIMAT0000825 | rno-miR-106b-5p | 366568 | Slc30a3 |
| MIMAT0000825 | rno-miR-106b-5p | 309035 | Dcun1d3 |
| MIMAT0000825 | rno-miR-106b-5p | 83842 | Crot |
| MIMAT0000825 | rno-miR-106b-5p | 83505 | St3gal5 |
| MIMAT0000825 | rno-miR-106b-5p | 501563 | Prkx |
| MIMAT0000825 | rno-miR-106b-5p | 305302 | Rasl11b |
| MIMAT0000825 | rno-miR-106b-5p | 494201 | Oas1e |
| MIMAT0000825 | rno-miR-106b-5p | 393087 | Pcdha5 |
| MIMAT0000825 | rno-miR-106b-5p | 313994 | Klf11 |
| MIMAT0000825 | rno-miR-106b-5p | 310448 | Igsf10 |
| MIMAT0000825 | rno-miR-106b-5p | 365963 | Lhx8 |
| MIMAT0000825 | rno-miR-106b-5p | 29525 | Pitpna |
| MIMAT0000825 | rno-miR-106b-5p | 304850 | Rnf2 |
| MIMAT0000825 | rno-miR-106b-5p | 65185 | Sult1c3 |
| MIMAT0000825 | rno-miR-106b-5p | 50677 | Ptpro |
| MIMAT0000825 | rno-miR-106b-5p | 29748 | Ppp3r1 |
| MIMAT0000825 | rno-miR-106b-5p | 315159 | Tob2 |
| MIMAT0000825 | rno-miR-106b-5p | 114090 | Egr2 |
| MIMAT0000825 | rno-miR-106b-5p | 29733 | S1pr1 |
| MIMAT0000825 | rno-miR-106b-5p | 171070 | Ptpn21 |
| MIMAT0000825 | rno-miR-106b-5p | 57300 | Aadac |
| MIMAT0000825 | rno-miR-106b-5p | 362061 | Cryz |
| MIMAT0000825 | rno-miR-106b-5p | 170845 | Ndel1 |
| MIMAT0000825 | rno-miR-106b-5p | 117186 | Sh3bp5 |
| MIMAT0000825 | rno-miR-106b-5p | 296851 | Pon2 |
| MIMAT0000825 | rno-miR-106b-5p | 286973 | Elavl2 |
| MIMAT0000825 | rno-miR-106b-5p | 64627 | Hist1h4b |
| MIMAT0000825 | rno-miR-106b-5p | 114246 | Trpv6 |
| MIMAT0000825 | rno-miR-106b-5p | 306695 | Zfp367 |
| MIMAT0000825 | rno-miR-106b-5p | 287765 | Ddx5 |
| MIMAT0000825 | rno-miR-106b-5p | 81655 | Dync1li2 |
| MIMAT0000825 | rno-miR-106b-5p | 500118 | LOC500118 |
| MIMAT0000825 | rno-miR-106b-5p | 65172 | Limk1 |
| MIMAT0000825 | rno-miR-106b-5p | 84481 | Arid4b |
| MIMAT0000825 | rno-miR-106b-5p | 299618 | Mknk2 |
| MIMAT0000825 | rno-miR-106b-5p | 25687 | Cald1 |
| MIMAT0000825 | rno-miR-106b-5p | 64044 | Casp8 |
| MIMAT0000825 | rno-miR-106b-5p | 308821 | Rab30 |
| MIMAT0000825 | rno-miR-106b-5p | 113894 | Sqstm1 |
| MIMAT0000825 | rno-miR-106b-5p | 393089 | Pcdha7 |
| MIMAT0000825 | rno-miR-106b-5p | 308890 | Btbd10 |
| MIMAT0000825 | rno-miR-106b-5p | 294322 | Pknox1 |
| MIMAT0000825 | rno-miR-106b-5p | 393091 | Pcdhac1 |
| MIMAT0000825 | rno-miR-106b-5p | 499856 | Fibin |
| MIMAT0000825 | rno-miR-106b-5p | 60562 | Stx6 |
| MIMAT0000825 | rno-miR-106b-5p | 298875 | Laptm4a |
| MIMAT0000825 | rno-miR-106b-5p | 363227 | Obfc2a |
| MIMAT0000825 | rno-miR-106b-5p | 29659 | P2rx4 |
| MIMAT0000825 | rno-miR-106b-5p | 114901 | Sorbs2 |
| MIMAT0000825 | rno-miR-106b-5p | 314906 | Kif5a |
| MIMAT0000825 | rno-miR-106b-5p | 296616 | Cercam |
| MIMAT0000825 | rno-miR-106b-5p | 303567 | Tmub2 |
| MIMAT0000825 | rno-miR-106b-5p | 360426 | Pla2g6 |
| MIMAT0000825 | rno-miR-106b-5p | 499331 | LOC499331 |
| MIMAT0000825 | rno-miR-106b-5p | 289307 | Tfb2m |
| MIMAT0000825 | rno-miR-106b-5p | 404781 | Iqsec3 |
| MIMAT0000825 | rno-miR-106b-5p | 54281 | Furin |
| MIMAT0000825 | rno-miR-106b-5p | 84550 | Slc24a2 |
| MIMAT0000825 | rno-miR-106b-5p | 25565 | Tle4 |
| MIMAT0000825 | rno-miR-106b-5p | 81758 | Rbl2 |
| MIMAT0000825 | rno-miR-106b-5p | 366507 | Lzic |
| MIMAT0000825 | rno-miR-106b-5p | 80881 | Serp1 |
| MIMAT0000825 | rno-miR-106b-5p | 29480 | Rgs4 |
| MIMAT0000825 | rno-miR-106b-5p | 29758 | St6galnac3 |
| MIMAT0000825 | rno-miR-106b-5p | 85426 | Slc5a7 |
| MIMAT0000825 | rno-miR-106b-5p | 117514 | Txnip |
| MIMAT0000825 | rno-miR-106b-5p | 25579 | Map3k12 |
| MIMAT0000825 | rno-miR-106b-5p | 294289 | Zbtb9 |
| MIMAT0000825 | rno-miR-106b-5p | 25095 | Ampd3 |
| MIMAT0000825 | rno-miR-106b-5p | 287828 | Hn1 |
| MIMAT0000825 | rno-miR-106b-5p | 288908 | Cc2d1a |
| MIMAT0000825 | rno-miR-106b-5p | 192152 | Tgoln1 |
| MIMAT0000825 | rno-miR-106b-5p | 83616 | Trim3 |
| MIMAT0000825 | rno-miR-106b-5p | 287543 | Tnfaip1 |
| MIMAT0000825 | rno-miR-106b-5p | 360549 | Plscr3 |
| MIMAT0000825 | rno-miR-106b-5p | 116742 | Pcdha13 |
| MIMAT0000825 | rno-miR-106b-5p | 362626 | RGD1359529 |
| MIMAT0000825 | rno-miR-106b-5p | 116596 | Map3k8 |
| MIMAT0000825 | rno-miR-106b-5p | 27080 | Hbp1 |
| MIMAT0000825 | rno-miR-106b-5p | 293886 | Cdc37l1 |
| MIMAT0000825 | rno-miR-106b-5p | 58853 | Nr4a3 |
| MIMAT0000825 | rno-miR-106b-5p | 114764 | Mapre1 |
| MIMAT0000825 | rno-miR-106b-5p | 361550 | Gramd1a |
| MIMAT0000825 | rno-miR-106b-5p | 311406 | Dusp2 |
| MIMAT0000825 | rno-miR-106b-5p | 117556 | Sv2b |
| MIMAT0000825 | rno-miR-106b-5p | 192280 | Ppp1r3b |
| MIMAT0000825 | rno-miR-106b-5p | 305816 | Ddhd1 |
| MIMAT0000825 | rno-miR-106b-5p | 298410 | Cmpk1 |
| MIMAT0000825 | rno-miR-106b-5p | 64189 | Pafah1b2 |
| MIMAT0000825 | rno-miR-106b-5p | 54230 | Btg3 |
| MIMAT0000825 | rno-miR-106b-5p | 298848 | Mapre3 |
| MIMAT0000825 | rno-miR-106b-5p | 117560 | Klf9 |
| MIMAT0000825 | rno-miR-106b-5p | 499653 | Gon4l |
| MIMAT0000830 | rno-miR-125b-5p | 293023 | Klhl25 |
| MIMAT0000830 | rno-miR-125b-5p | 83721 | Dvl1 |
| MIMAT0000830 | rno-miR-125b-5p | 246142 | Bmf |
| MIMAT0000830 | rno-miR-125b-5p | 25073 | Scarb1 |
| MIMAT0000830 | rno-miR-125b-5p | 292657 | Slc1a5 |
| MIMAT0000830 | rno-miR-125b-5p | 362261 | Lpin3 |
| MIMAT0000830 | rno-miR-125b-5p | 29619 | Btg2 |
| MIMAT0000830 | rno-miR-125b-5p | 116502 | Bak1 |
| MIMAT0000830 | rno-miR-125b-5p | 314442 | Wars |
| MIMAT0000830 | rno-miR-125b-5p | 257648 | Cacna1b |
| MIMAT0000830 | rno-miR-125b-5p | 245918 | Cgref1 |
| MIMAT0000830 | rno-miR-125b-5p | 494344 | Ier2 |
| MIMAT0000830 | rno-miR-125b-5p | 83588 | Kcns3 |
| MIMAT0000830 | rno-miR-125b-5p | 64442 | St3gal2 |
| MIMAT0000830 | rno-miR-125b-5p | 171082 | Atp5g2 |
| MIMAT0000830 | rno-miR-125b-5p | 117107 | Zbtb7a |
| MIMAT0000830 | rno-miR-125b-5p | 83764 | Flot2 |
| MIMAT0000830 | rno-miR-125b-5p | 363448 | Dynlt3 |
| MIMAT0000830 | rno-miR-125b-5p | 313878 | Galnt14 |
| MIMAT0000830 | rno-miR-125b-5p | 25217 | Tapbp |
| MIMAT0000830 | rno-miR-125b-5p | 25455 | Ggh |
| MIMAT0000830 | rno-miR-125b-5p | 362334 | Tmem140 |
| MIMAT0000830 | rno-miR-125b-5p | 192204 | Ehd4 |
| MIMAT0000830 | rno-miR-125b-5p | 79011 | Camkv |
| MIMAT0000830 | rno-miR-125b-5p | 312727 | Tspan11 |
| MIMAT0000830 | rno-miR-125b-5p | 25734 | Hck |
| MIMAT0000830 | rno-miR-125b-5p | 29513 | Mapk13 |
| MIMAT0000830 | rno-miR-125b-5p | 84396 | Atp1b4 |
| MIMAT0000830 | rno-miR-125b-5p | 116589 | Serpinb5 |
| MIMAT0000830 | rno-miR-125b-5p | 498266 | Blzf1 |
| MIMAT0000830 | rno-miR-125b-5p | 312135 | Tmem168 |
| MIMAT0000830 | rno-miR-125b-5p | 282634 | Pxmp4 |
| MIMAT0000830 | rno-miR-125b-5p | 89814 | Tnfsf4 |
| MIMAT0000830 | rno-miR-125b-5p | 257647 | Slc35a4 |
| MIMAT0000830 | rno-miR-125b-5p | 295645 | Slc4a10 |
| MIMAT0000830 | rno-miR-125b-5p | 29606 | Pcsk7 |
| MIMAT0000830 | rno-miR-125b-5p | 81008 | Itga7 |
| MIMAT0000830 | rno-miR-125b-5p | 171402 | Elovl6 |
| MIMAT0000830 | rno-miR-125b-5p | 362987 | Rabl2b |
| MIMAT0000830 | rno-miR-125b-5p | 84424 | Tle3 |
| MIMAT0000830 | rno-miR-125b-5p | 305795 | Abhd6 |
| MIMAT0000830 | rno-miR-125b-5p | 359959 | Rhebl1 |
| MIMAT0000830 | rno-miR-125b-5p | 58958 | Nup210 |
| MIMAT0000830 | rno-miR-125b-5p | 83510 | Lypla2 |
| MIMAT0000830 | rno-miR-125b-5p | 304860 | Npl |
| MIMAT0000830 | rno-miR-125b-5p | 117055 | Gjb4 |
| MIMAT0000830 | rno-miR-125b-5p | 314648 | Ncln |
| MIMAT0000830 | rno-miR-125b-5p | 58982 | Ncan |
| MIMAT0000830 | rno-miR-125b-5p | 360457 | Figf |
| MIMAT0000830 | rno-miR-125b-5p | 60666 | Gpd1 |
| MIMAT0000830 | rno-miR-125b-5p | 287156 | Rhot2 |
| MIMAT0000830 | rno-miR-125b-5p | 305482 | Mtmr3 |
| MIMAT0000830 | rno-miR-125b-5p | 497934 | LOC497934 |
| MIMAT0000830 | rno-miR-125b-5p | 85265 | Jub |
| MIMAT0000830 | rno-miR-125b-5p | 24873 | Vdr |
| MIMAT0000830 | rno-miR-125b-5p | 304851 | RGD1307890 |
| MIMAT0000830 | rno-miR-125b-5p | 81919 | Fut1 |
| MIMAT0000830 | rno-miR-125b-5p | 296318 | Ndrg3 |
| MIMAT0000830 | rno-miR-125b-5p | 362011 | Tmem77 |
| MIMAT0000830 | rno-miR-125b-5p | 361791 | Nrm |
| MIMAT0000830 | rno-miR-125b-5p | 499672 | Cdc42se1 |
| MIMAT0000830 | rno-miR-125b-5p | 116721 | Abcc5 |
| MIMAT0000830 | rno-miR-125b-5p | 64191 | Dhcr7 |
| MIMAT0000830 | rno-miR-125b-5p | 362364 | RGD1310827 |
| MIMAT0000830 | rno-miR-125b-5p | 360834 | Vps4b |
| MIMAT0000830 | rno-miR-125b-5p | 295347 | Rap1a |
| MIMAT0000830 | rno-miR-125b-5p | 116486 | Sec14l2 |
| MIMAT0000830 | rno-miR-125b-5p | 362715 | Dtnb |
| MIMAT0000830 | rno-miR-125b-5p | 25155 | Syk |
| MIMAT0000830 | rno-miR-125b-5p | 499108 | Sertad3 |
| MIMAT0000830 | rno-miR-125b-5p | 171152 | Taf9b |
| MIMAT0000830 | rno-miR-125b-5p | 619550 | Gpr153 |
| MIMAT0000830 | rno-miR-125b-5p | 83469 | Lrp4 |
| MIMAT0000830 | rno-miR-125b-5p | 293673 | Eif1ad |
| MIMAT0000830 | rno-miR-125b-5p | 83627 | Galnt5 |
| MIMAT0000830 | rno-miR-125b-5p | 156275 | Ggt7 |
| MIMAT0000830 | rno-miR-125b-5p | 315114 | C1qtnf6 |
| MIMAT0000830 | rno-miR-125b-5p | 406868 | G4 |
| MIMAT0000830 | rno-miR-125b-5p | 307833 | RGD1307799 |
| MIMAT0000830 | rno-miR-125b-5p | 300015 | RGD1359378 |
| MIMAT0000830 | rno-miR-125b-5p | 25125 | Stat3 |
| MIMAT0000830 | rno-miR-125b-5p | 116636 | Eif4ebp1 |
| MIMAT0000830 | rno-miR-125b-5p | 303803 | Klhl24 |
| MIMAT0000830 | rno-miR-125b-5p | 286939 | Asam |
| MIMAT0000830 | rno-miR-125b-5p | 297432 | Abtb1 |
| MIMAT0000830 | rno-miR-125b-5p | 29659 | P2rx4 |
| MIMAT0000830 | rno-miR-125b-5p | 309457 | Pcgf6 |
| MIMAT0000830 | rno-miR-125b-5p | 64304 | Acads |
| MIMAT0000830 | rno-miR-125b-5p | 171044 | Sstr3 |
| MIMAT0000830 | rno-miR-125b-5p | 311547 | Foxs1 |
| MIMAT0000830 | rno-miR-125b-5p | 313235 | Coro2a |
| MIMAT0000830 | rno-miR-125b-5p | 29745 | Sema4f |
| MIMAT0000830 | rno-miR-125b-5p | 361757 | Opalin |
| MIMAT0000830 | rno-miR-125b-5p | 294289 | Zbtb9 |
| MIMAT0000830 | rno-miR-125b-5p | 293668 | Brms1 |
| MIMAT0000830 | rno-miR-125b-5p | 192152 | Tgoln1 |
| MIMAT0000830 | rno-miR-125b-5p | 24697 | Ptpn1 |
| MIMAT0000830 | rno-miR-125b-5p | 498999 | Ahrr |
| MIMAT0000830 | rno-miR-125b-5p | 311429 | RGD1311267 |
| MIMAT0000830 | rno-miR-125b-5p | 366227 | RGD1308874 |
| MIMAT0000830 | rno-miR-125b-5p | 64467 | Entpd2 |
| MIMAT0000830 | rno-miR-125b-5p | 314323 | Flvcr2 |
| MIMAT0000830 | rno-miR-125b-5p | 295322 | Vtcn1 |
| MIMAT0000830 | rno-miR-125b-5p | 65272 | Kcnk10 |
| MIMAT0000830 | rno-miR-125b-5p | 308976 | Jmjd5 |
| MIMAT0000830 | rno-miR-125b-5p | 85428 | Rhoq |
| MIMAT0000830 | rno-miR-125b-5p | 363521 | Taz |
| MIMAT0000830 | rno-miR-125b-5p | 362112 | Tor2a |
| MIMAT0000830 | rno-miR-125b-5p | 282819 | Ppif |
| MIMAT0000830 | rno-miR-125b-5p | 300095 | Srebf2 |
| MIMAT0000836 | rno-miR-130a-3p | 25524 | Psap |
| MIMAT0000836 | rno-miR-130a-3p | 360571 | Rab34 |
| MIMAT0000836 | rno-miR-130a-3p | 25664 | Pparg |
| MIMAT0000836 | rno-miR-130a-3p | 83526 | Atrn |
| MIMAT0000836 | rno-miR-130a-3p | 64517 | Thop1 |
| MIMAT0000836 | rno-miR-130a-3p | 29618 | Btg1 |
| MIMAT0000836 | rno-miR-130a-3p | 282834 | Vps24 |
| MIMAT0000836 | rno-miR-130a-3p | 246310 | Arfgap1 |
| MIMAT0000836 | rno-miR-130a-3p | 65183 | Aldh3a2 |
| MIMAT0000836 | rno-miR-130a-3p | 297337 | Rnf181 |
| MIMAT0000836 | rno-miR-130a-3p | 404280 | Mid1ip1 |
| MIMAT0000836 | rno-miR-130a-3p | 171396 | Sulf1 |
| MIMAT0000836 | rno-miR-130a-3p | 81809 | Tgfb2 |
| MIMAT0000836 | rno-miR-130a-3p | 308937 | Wee1 |
| MIMAT0000836 | rno-miR-130a-3p | 246232 | Uxs1 |
| MIMAT0000836 | rno-miR-130a-3p | 58839 | Nrbf2 |
| MIMAT0000836 | rno-miR-130a-3p | 493574 | Ispd |
| MIMAT0000836 | rno-miR-130a-3p | 25469 | Kcna4 |
| MIMAT0000836 | rno-miR-130a-3p | 290775 | Vps37a |
| MIMAT0000836 | rno-miR-130a-3p | 266680 | St18 |
| MIMAT0000836 | rno-miR-130a-3p | 84010 | Dll1 |
| MIMAT0000836 | rno-miR-130a-3p | 54241 | Cltc |
| MIMAT0000836 | rno-miR-130a-3p | 305434 | Jakmip1 |
| MIMAT0000836 | rno-miR-130a-3p | 290947 | Mtrr |
| MIMAT0000836 | rno-miR-130a-3p | 113976 | Acsl4 |
| MIMAT0000836 | rno-miR-130a-3p | 25708 | Ucp3 |
| MIMAT0000836 | rno-miR-130a-3p | 29463 | Ptp4a1 |
| MIMAT0000836 | rno-miR-130a-3p | 494320 | Ccdc8 |
| MIMAT0000836 | rno-miR-130a-3p | 24783 | Slc9a2 |
| MIMAT0000836 | rno-miR-130a-3p | 25570 | Trhr |
| MIMAT0000836 | rno-miR-130a-3p | 170914 | Nap1l3 |
| MIMAT0000836 | rno-miR-130a-3p | 260323 | Snx27 |
| MIMAT0000836 | rno-miR-130a-3p | 81925 | Cds1 |
| MIMAT0000836 | rno-miR-130a-3p | 24392 | Gja1 |
| MIMAT0000836 | rno-miR-130a-3p | 58967 | Sfmbt1 |
| MIMAT0000836 | rno-miR-130a-3p | 117553 | Uba3 |
| MIMAT0000836 | rno-miR-130a-3p | 24908 | Dnajb9 |
| MIMAT0000836 | rno-miR-130a-3p | 24508 | Irf1 |
| MIMAT0000836 | rno-miR-130a-3p | 304766 | Ubxn4 |
| MIMAT0000836 | rno-miR-130a-3p | 295217 | Snapap |
| MIMAT0000836 | rno-miR-130a-3p | 313843 | Galm |
| MIMAT0000836 | rno-miR-130a-3p | 56064 | Nptn |
| MIMAT0000836 | rno-miR-130a-3p | 282835 | Wrnip1 |
| MIMAT0000836 | rno-miR-130a-3p | 29187 | Cd69 |
| MIMAT0000836 | rno-miR-130a-3p | 316249 | Enpp5 |
| MIMAT0000836 | rno-miR-130a-3p | 24948 | Fmr1 |
| MIMAT0000836 | rno-miR-130a-3p | 296741 | Fastk |
| MIMAT0000836 | rno-miR-130a-3p | 25751 | Dnm2 |
| MIMAT0000836 | rno-miR-130a-3p | 116721 | Abcc5 |
| MIMAT0000836 | rno-miR-130a-3p | 29279 | Meox2 |
| MIMAT0000836 | rno-miR-130a-3p | 360950 | Wdr1 |
| MIMAT0000836 | rno-miR-130a-3p | 313588 | Snip1 |
| MIMAT0000836 | rno-miR-130a-3p | 303924 | Upk1b |
| MIMAT0000836 | rno-miR-130a-3p | 65201 | Clip1 |
| MIMAT0000836 | rno-miR-130a-3p | 308589 | Sphk2 |
| MIMAT0000836 | rno-miR-130a-3p | 84014 | Ralbp1 |
| MIMAT0000836 | rno-miR-130a-3p | 308051 | Rnf146 |
| MIMAT0000836 | rno-miR-130a-3p | 117596 | Atp6v1b2 |
| MIMAT0000836 | rno-miR-130a-3p | 29733 | S1pr1 |
| MIMAT0000836 | rno-miR-130a-3p | 366734 | Bag5 |
| MIMAT0000836 | rno-miR-130a-3p | 171113 | Blcap |
| MIMAT0000836 | rno-miR-130a-3p | 29721 | Hivep2 |
| MIMAT0000836 | rno-miR-130a-3p | 363869 | Ubl3 |
| MIMAT0000836 | rno-miR-130a-3p | 361391 | Cbfb |
| MIMAT0000836 | rno-miR-130a-3p | 170845 | Ndel1 |
| MIMAT0000836 | rno-miR-130a-3p | 310848 | Cyp2u1 |
| MIMAT0000836 | rno-miR-130a-3p | 83469 | Lrp4 |
| MIMAT0000836 | rno-miR-130a-3p | 81655 | Dync1li2 |
| MIMAT0000836 | rno-miR-130a-3p | 246334 | Tp63 |
| MIMAT0000836 | rno-miR-130a-3p | 295934 | Chst1 |
| MIMAT0000836 | rno-miR-130a-3p | 360915 | Cops4 |
| MIMAT0000836 | rno-miR-130a-3p | 84481 | Arid4b |
| MIMAT0000836 | rno-miR-130a-3p | 24465 | Hprt1 |
| MIMAT0000836 | rno-miR-130a-3p | 361285 | Mllt10 |
| MIMAT0000836 | rno-miR-130a-3p | 308890 | Btbd10 |
| MIMAT0000836 | rno-miR-130a-3p | 499856 | Fibin |
| MIMAT0000836 | rno-miR-130a-3p | 60562 | Stx6 |
| MIMAT0000836 | rno-miR-130a-3p | 298875 | Laptm4a |
| MIMAT0000836 | rno-miR-130a-3p | 60382 | Arfip1 |
| MIMAT0000836 | rno-miR-130a-3p | 25547 | St8sia3 |
| MIMAT0000836 | rno-miR-130a-3p | 362849 | March2 |
| MIMAT0000836 | rno-miR-130a-3p | 84550 | Slc24a2 |
| MIMAT0000836 | rno-miR-130a-3p | 366507 | Lzic |
| MIMAT0000836 | rno-miR-130a-3p | 297498 | Crbn |
| MIMAT0000836 | rno-miR-130a-3p | 360502 | Itfg3 |
| MIMAT0000836 | rno-miR-130a-3p | 170908 | Tesk2 |
| MIMAT0000836 | rno-miR-130a-3p | 498749 | Ttrap |
| MIMAT0000836 | rno-miR-130a-3p | 171501 | Rnf38 |
| MIMAT0000836 | rno-miR-130a-3p | 25579 | Map3k12 |
| MIMAT0000836 | rno-miR-130a-3p | 310999 | Plekhg5 |
| MIMAT0000836 | rno-miR-130a-3p | 25095 | Ampd3 |
| MIMAT0000836 | rno-miR-130a-3p | 60586 | Clcn4-2 |
| MIMAT0000836 | rno-miR-130a-3p | 83616 | Trim3 |
| MIMAT0000836 | rno-miR-130a-3p | 25112 | Gadd45a |
| MIMAT0000836 | rno-miR-130a-3p | 116596 | Map3k8 |
| MIMAT0000836 | rno-miR-130a-3p | 27080 | Hbp1 |
| MIMAT0000836 | rno-miR-130a-3p | 361110 | Tmem110 |
| MIMAT0000836 | rno-miR-130a-3p | 25288 | Acsl1 |
| MIMAT0000836 | rno-miR-130a-3p | 298410 | Cmpk1 |
| MIMAT0000836 | rno-miR-130a-3p | 25262 | Itpr1 |
| MIMAT0000836 | rno-miR-130a-3p | 59329 | Snf1lk |
| MIMAT0000836 | rno-miR-130a-3p | 83833 | Smarcd2 |
| MIMAT0000836 | rno-miR-130a-3p | 246776 | Filip1 |
| MIMAT0000836 | rno-miR-130a-3p | 499653 | Gon4l |
| MIMAT0000836 | rno-miR-130a-3p | 156873 | Kcnk15 |
| MIMAT0000836 | rno-miR-130a-3p | 207123 | RGD621098 |
| MIMAT0000846 | rno-miR-141-3p | 24483 | Igf2 |
| MIMAT0000846 | rno-miR-141-3p | 171060 | Il13ra2 |
| MIMAT0000846 | rno-miR-141-3p | 312903 | Tram1 |
| MIMAT0000846 | rno-miR-141-3p | 117104 | Ppp2r2a |
| MIMAT0000846 | rno-miR-141-3p | 304543 | Mlec |
| MIMAT0000846 | rno-miR-141-3p | 84487 | Slc17a6 |
| MIMAT0000846 | rno-miR-141-3p | 365493 | RGD1308127 |
| MIMAT0000846 | rno-miR-141-3p | 288233 | Wrb |
| MIMAT0000846 | rno-miR-141-3p | 171121 | Ppp6c |
| MIMAT0000846 | rno-miR-141-3p | 298792 | Ypel5 |
| MIMAT0000846 | rno-miR-141-3p | 25514 | Lypla1 |
| MIMAT0000846 | rno-miR-141-3p | 497815 | Nrcam |
| MIMAT0000846 | rno-miR-141-3p | 293098 | Tmem135 |
| MIMAT0000846 | rno-miR-141-3p | 362750 | Atl1 |
| MIMAT0000846 | rno-miR-141-3p | 25694 | Has2 |
| MIMAT0000846 | rno-miR-141-3p | 29170 | Aqp6 |
| MIMAT0000846 | rno-miR-141-3p | 360854 | Arpc5 |
| MIMAT0000846 | rno-miR-141-3p | 81809 | Tgfb2 |
| MIMAT0000846 | rno-miR-141-3p | 299857 | Shmt2 |
| MIMAT0000846 | rno-miR-141-3p | 83580 | Thbd |
| MIMAT0000846 | rno-miR-141-3p | 296731 | Nub1 |
| MIMAT0000846 | rno-miR-141-3p | 114561 | Pitpnb |
| MIMAT0000846 | rno-miR-141-3p | 363545 | Nat15 |
| MIMAT0000846 | rno-miR-141-3p | 24516 | Jun |
| MIMAT0000846 | rno-miR-141-3p | 170956 | Yt521 |
| MIMAT0000846 | rno-miR-141-3p | 29637 | Hmgcs1 |
| MIMAT0000846 | rno-miR-141-3p | 361944 | Elf2 |
| MIMAT0000846 | rno-miR-141-3p | 83534 | Tpp1 |
| MIMAT0000846 | rno-miR-141-3p | 498160 | Zkscan1 |
| MIMAT0000846 | rno-miR-141-3p | 114216 | S100a3 |
| MIMAT0000846 | rno-miR-141-3p | 306809 | Bicd2 |
| MIMAT0000846 | rno-miR-141-3p | 84587 | Plcl1 |
| MIMAT0000846 | rno-miR-141-3p | 24856 | Ttr |
| MIMAT0000846 | rno-miR-141-3p | 312135 | Tmem168 |
| MIMAT0000846 | rno-miR-141-3p | 306817 | Dek |
| MIMAT0000846 | rno-miR-141-3p | 65190 | Rsad2 |
| MIMAT0000846 | rno-miR-141-3p | 291078 | Prpf4b |
| MIMAT0000846 | rno-miR-141-3p | 29463 | Ptp4a1 |
| MIMAT0000846 | rno-miR-141-3p | 114021 | Ebna1bp2 |
| MIMAT0000846 | rno-miR-141-3p | 114514 | Clasp2 |
| MIMAT0000846 | rno-miR-141-3p | 79433 | Myh10 |
| MIMAT0000846 | rno-miR-141-3p | 29345 | Serpinh1 |
| MIMAT0000846 | rno-miR-141-3p | 170704 | Hrh4 |
| MIMAT0000846 | rno-miR-141-3p | 24672 | Ppp2ca |
| MIMAT0000846 | rno-miR-141-3p | 116506 | Calcr |
| MIMAT0000846 | rno-miR-141-3p | 116509 | Slc6a9 |
| MIMAT0000846 | rno-miR-141-3p | 26759 | Acot7 |
| MIMAT0000846 | rno-miR-141-3p | 260323 | Snx27 |
| MIMAT0000846 | rno-miR-141-3p | 25389 | Atf3 |
| MIMAT0000846 | rno-miR-141-3p | 360868 | Sft2d2 |
| MIMAT0000846 | rno-miR-141-3p | 369016 | Myadm |
| MIMAT0000846 | rno-miR-141-3p | 59265 | Phlpp1 |
| MIMAT0000846 | rno-miR-141-3p | 24413 | Nr3c1 |
| MIMAT0000846 | rno-miR-141-3p | 140670 | Ap2b1 |
| MIMAT0000846 | rno-miR-141-3p | 245959 | Slc25a3 |
| MIMAT0000846 | rno-miR-141-3p | 65137 | Ruvbl1 |
| MIMAT0000846 | rno-miR-141-3p | 191575 | Nme1 |
| MIMAT0000846 | rno-miR-141-3p | 29564 | Dync1i1 |
| MIMAT0000846 | rno-miR-141-3p | 24188 | Aldh1a1 |
| MIMAT0000846 | rno-miR-141-3p | 361810 | Fkbp5 |
| MIMAT0000846 | rno-miR-141-3p | 311346 | Lrrc57 |
| MIMAT0000846 | rno-miR-141-3p | 311846 | Lrrc8a |
| MIMAT0000846 | rno-miR-141-3p | 93667 | Olfm1 |
| MIMAT0000846 | rno-miR-141-3p | 83842 | Crot |
| MIMAT0000846 | rno-miR-141-3p | 24942 | Chm |
| MIMAT0000846 | rno-miR-141-3p | 291948 | Pgrmc1 |
| MIMAT0000846 | rno-miR-141-3p | 29735 | Slc16a7 |
| MIMAT0000846 | rno-miR-141-3p | 315608 | Ube4a |
| MIMAT0000846 | rno-miR-141-3p | 313588 | Snip1 |
| MIMAT0000846 | rno-miR-141-3p | 56010 | Ywhag |
| MIMAT0000846 | rno-miR-141-3p | 25054 | Ntrk2 |
| MIMAT0000846 | rno-miR-141-3p | 171458 | Ipmk |
| MIMAT0000846 | rno-miR-141-3p | 296478 | Zgpat |
| MIMAT0000846 | rno-miR-141-3p | 302669 | Car5b |
| MIMAT0000846 | rno-miR-141-3p | 25187 | Htr2c |
| MIMAT0000846 | rno-miR-141-3p | 117596 | Atp6v1b2 |
| MIMAT0000846 | rno-miR-141-3p | 300886 | Mthfs |
| MIMAT0000846 | rno-miR-141-3p | 500941 | MGC105560 |
| MIMAT0000846 | rno-miR-141-3p | 499602 | LOC499602 |
| MIMAT0000846 | rno-miR-141-3p | 29149 | Strn |
| MIMAT0000846 | rno-miR-141-3p | 117559 | Sv2a |
| MIMAT0000846 | rno-miR-141-3p | 362061 | Cryz |
| MIMAT0000846 | rno-miR-141-3p | 252916 | Rab38 |
| MIMAT0000846 | rno-miR-141-3p | 296315 | RGD1307752 |
| MIMAT0000846 | rno-miR-141-3p | 286973 | Elavl2 |
| MIMAT0000846 | rno-miR-141-3p | 24918 | Stat5a |
| MIMAT0000846 | rno-miR-141-3p | 64551 | Sept7 |
| MIMAT0000846 | rno-miR-141-3p | 312495 | Cyp26b1 |
| MIMAT0000846 | rno-miR-141-3p | 170906 | Zdhhc7 |
| MIMAT0000846 | rno-miR-141-3p | 54398 | Ppt2 |
| MIMAT0000846 | rno-miR-141-3p | 307505 | Ctnna1 |
| MIMAT0000846 | rno-miR-141-3p | 287765 | Ddx5 |
| MIMAT0000846 | rno-miR-141-3p | 246334 | Tp63 |
| MIMAT0000846 | rno-miR-141-3p | 25615 | Sdc2 |
| MIMAT0000846 | rno-miR-141-3p | 315741 | Paqr5 |
| MIMAT0000846 | rno-miR-141-3p | 54284 | Pitx2 |
| MIMAT0000846 | rno-miR-141-3p | 64200 | Hnrnpf |
| MIMAT0000846 | rno-miR-141-3p | 291534 | Rnmt |
| MIMAT0000846 | rno-miR-141-3p | 116482 | Sacm1l |
| MIMAT0000846 | rno-miR-141-3p | 298943 | Bcap29 |
| MIMAT0000846 | rno-miR-141-3p | 306720 | Ctsm |
| MIMAT0000846 | rno-miR-141-3p | 140941 | Siah1a |
| MIMAT0000846 | rno-miR-141-3p | 65196 | B4galt6 |
| MIMAT0000846 | rno-miR-141-3p | 81684 | Mipep |
| MIMAT0000846 | rno-miR-141-3p | 116744 | Lpar1 |
| MIMAT0000846 | rno-miR-141-3p | 29544 | Tspyl1 |
| MIMAT0000846 | rno-miR-141-3p | 116551 | Pdk1 |
| MIMAT0000846 | rno-miR-141-3p | 60443 | Epn2 |
| MIMAT0000846 | rno-miR-141-3p | 60562 | Stx6 |
| MIMAT0000846 | rno-miR-141-3p | 317385 | Fus |
| MIMAT0000846 | rno-miR-141-3p | 290032 | Fam12b |
| MIMAT0000846 | rno-miR-141-3p | 297123 | Fkbp9 |
| MIMAT0000846 | rno-miR-141-3p | 64469 | Slc30a4 |
| MIMAT0000846 | rno-miR-141-3p | 29592 | Bcat1 |
| MIMAT0000846 | rno-miR-141-3p | 83685 | Capn6 |
| MIMAT0000846 | rno-miR-141-3p | 25558 | Stxbp1 |
| MIMAT0000846 | rno-miR-141-3p | 50655 | Aco1 |
| MIMAT0000846 | rno-miR-141-3p | 58976 | Slc30a1 |
| MIMAT0000846 | rno-miR-141-3p | 304791 | Ripk5 |
| MIMAT0000846 | rno-miR-141-3p | 117514 | Txnip |
| MIMAT0000846 | rno-miR-141-3p | 289881 | Dr1 |
| MIMAT0000846 | rno-miR-141-3p | 64152 | Chp |
| MIMAT0000846 | rno-miR-141-3p | 84352 | Col1a2 |
| MIMAT0000846 | rno-miR-141-3p | 60586 | Clcn4-2 |
| MIMAT0000846 | rno-miR-141-3p | 29657 | Arntl |
| MIMAT0000846 | rno-miR-141-3p | 171517 | Gpc2 |
| MIMAT0000846 | rno-miR-141-3p | 66025 | Lpar3 |
| MIMAT0000846 | rno-miR-141-3p | 79119 | Arf2 |
| MIMAT0000846 | rno-miR-141-3p | 289883 | Tmed5 |
| MIMAT0000846 | rno-miR-141-3p | 24184 | Ak2 |
| MIMAT0000846 | rno-miR-141-3p | 361110 | Tmem110 |
| MIMAT0000846 | rno-miR-141-3p | 361442 | Sipa1l2 |
| MIMAT0000846 | rno-miR-141-3p | 300687 | Bud13 |
| MIMAT0000846 | rno-miR-141-3p | 497757 | Gucy1a3 |
| MIMAT0000846 | rno-miR-141-3p | 24831 | Thrb |
| MIMAT0000846 | rno-miR-141-3p | 25110 | Klrd1 |
| MIMAT0000846 | rno-miR-141-3p | 498564 | Itgbl1 |
| MIMAT0000846 | rno-miR-141-3p | 116668 | Myt1l |
| MIMAT0000858 | rno-miR-181a-5p | 298906 | Pqlc3 |
| MIMAT0000858 | rno-miR-181a-5p | 25524 | Psap |
| MIMAT0000858 | rno-miR-181a-5p | 393092 | Pcdhac2 |
| MIMAT0000858 | rno-miR-181a-5p | 314587 | Zfp472 |
| MIMAT0000858 | rno-miR-181a-5p | 362320 | Fam133b |
| MIMAT0000858 | rno-miR-181a-5p | 25306 | Cpd |
| MIMAT0000858 | rno-miR-181a-5p | 365355 | Rras2 |
| MIMAT0000858 | rno-miR-181a-5p | 25358 | Timp3 |
| MIMAT0000858 | rno-miR-181a-5p | 29642 | Slc38a2 |
| MIMAT0000858 | rno-miR-181a-5p | 116778 | Pcdha10 |
| MIMAT0000858 | rno-miR-181a-5p | 305910 | Pspc1 |
| MIMAT0000858 | rno-miR-181a-5p | 171160 | Hopx |
| MIMAT0000858 | rno-miR-181a-5p | 54193 | Pbsn |
| MIMAT0000858 | rno-miR-181a-5p | 25549 | Slc18a2 |
| MIMAT0000858 | rno-miR-181a-5p | 24835 | Tnf |
| MIMAT0000858 | rno-miR-181a-5p | 295401 | Lppr4 |
| MIMAT0000858 | rno-miR-181a-5p | 394223 | Pcdha11 |
| MIMAT0000858 | rno-miR-181a-5p | 366602 | Tspan13 |
| MIMAT0000858 | rno-miR-181a-5p | 66030 | Synpr |
| MIMAT0000858 | rno-miR-181a-5p | 170538 | Prkcd |
| MIMAT0000858 | rno-miR-181a-5p | 192256 | Pja2 |
| MIMAT0000858 | rno-miR-181a-5p | 116781 | Pcdha8 |
| MIMAT0000858 | rno-miR-181a-5p | 393086 | Pcdha2 |
| MIMAT0000858 | rno-miR-181a-5p | 170851 | Map2k1 |
| MIMAT0000858 | rno-miR-181a-5p | 361565 | Vrk3 |
| MIMAT0000858 | rno-miR-181a-5p | 50938 | Cdon |
| MIMAT0000858 | rno-miR-181a-5p | 83712 | Rbbp7 |
| MIMAT0000858 | rno-miR-181a-5p | 25694 | Has2 |
| MIMAT0000858 | rno-miR-181a-5p | 362912 | Derl1 |
| MIMAT0000858 | rno-miR-181a-5p | 393085 | Pcdha1 |
| MIMAT0000858 | rno-miR-181a-5p | 116741 | Pcdha4 |
| MIMAT0000858 | rno-miR-181a-5p | 116600 | Cacnb2 |
| MIMAT0000858 | rno-miR-181a-5p | 297109 | MGC95152 |
| MIMAT0000858 | rno-miR-181a-5p | 313840 | Fam82a1 |
| MIMAT0000858 | rno-miR-181a-5p | 500110 | Znf467 |
| MIMAT0000858 | rno-miR-181a-5p | 58954 | Klf6 |
| MIMAT0000858 | rno-miR-181a-5p | 192204 | Ehd4 |
| MIMAT0000858 | rno-miR-181a-5p | 85497 | Klf15 |
| MIMAT0000858 | rno-miR-181a-5p | 362862 | Tra1 |
| MIMAT0000858 | rno-miR-181a-5p | 116779 | Pcdha12 |
| MIMAT0000858 | rno-miR-181a-5p | 406165 | Agpat1 |
| MIMAT0000858 | rno-miR-181a-5p | 25469 | Kcna4 |
| MIMAT0000858 | rno-miR-181a-5p | 50566 | Grem1 |
| MIMAT0000858 | rno-miR-181a-5p | 63840 | Per2 |
| MIMAT0000858 | rno-miR-181a-5p | 25341 | Tnfrsf11b |
| MIMAT0000858 | rno-miR-181a-5p | 286906 | Pdia6 |
| MIMAT0000858 | rno-miR-181a-5p | 116780 | Pcdha3 |
| MIMAT0000858 | rno-miR-181a-5p | 305419 | Pi4k2b |
| MIMAT0000858 | rno-miR-181a-5p | 24932 | Cd4 |
| MIMAT0000858 | rno-miR-181a-5p | 84586 | Fgl2 |
| MIMAT0000858 | rno-miR-181a-5p | 311872 | Zbtb43 |
| MIMAT0000858 | rno-miR-181a-5p | 29140 | Snn |
| MIMAT0000858 | rno-miR-181a-5p | 364403 | Blk |
| MIMAT0000858 | rno-miR-181a-5p | 81816 | Ube2b |
| MIMAT0000858 | rno-miR-181a-5p | 312135 | Tmem168 |
| MIMAT0000858 | rno-miR-181a-5p | 24356 | Ets1 |
| MIMAT0000858 | rno-miR-181a-5p | 362556 | Ttc4 |
| MIMAT0000858 | rno-miR-181a-5p | 315664 | Kdelc2 |
| MIMAT0000858 | rno-miR-181a-5p | 300721 | Dnaja4 |
| MIMAT0000858 | rno-miR-181a-5p | 363283 | Cops8 |
| MIMAT0000858 | rno-miR-181a-5p | 29705 | Gabra1 |
| MIMAT0000858 | rno-miR-181a-5p | 113976 | Acsl4 |
| MIMAT0000858 | rno-miR-181a-5p | 303926 | Igsf11 |
| MIMAT0000858 | rno-miR-181a-5p | 363026 | Carm1 |
| MIMAT0000858 | rno-miR-181a-5p | 81516 | Smad7 |
| MIMAT0000858 | rno-miR-181a-5p | 171109 | Dusp5 |
| MIMAT0000858 | rno-miR-181a-5p | 246074 | Scd1 |
| MIMAT0000858 | rno-miR-181a-5p | 360853 | Uchl5 |
| MIMAT0000858 | rno-miR-181a-5p | 314949 | Rad21 |
| MIMAT0000858 | rno-miR-181a-5p | 25650 | Atp1b1 |
| MIMAT0000858 | rno-miR-181a-5p | 393088 | Pcdha6 |
| MIMAT0000858 | rno-miR-181a-5p | 295674 | Plekha3 |
| MIMAT0000858 | rno-miR-181a-5p | 79252 | Adamts1 |
| MIMAT0000858 | rno-miR-181a-5p | 81707 | Mmp14 |
| MIMAT0000858 | rno-miR-181a-5p | 24413 | Nr3c1 |
| MIMAT0000858 | rno-miR-181a-5p | 303923 | B4galt4 |
| MIMAT0000858 | rno-miR-181a-5p | 24831 | Thrb |
| MIMAT0000858 | rno-miR-181a-5p | 298075 | Ncbp1 |
| MIMAT0000858 | rno-miR-181a-5p | 59303 | Tmem33 |
| MIMAT0000858 | rno-miR-181a-5p | 60341 | Camkk1 |
| MIMAT0000858 | rno-miR-181a-5p | 50572 | Slco1a1 |
| MIMAT0000858 | rno-miR-181a-5p | 290527 | Anxa11 |
| MIMAT0000858 | rno-miR-181a-5p | 291259 | Zmynd11 |
| MIMAT0000858 | rno-miR-181a-5p | 362778 | RGD1308470 |
| MIMAT0000858 | rno-miR-181a-5p | 29179 | Syn2 |
| MIMAT0000858 | rno-miR-181a-5p | 245920 | Cxcl10 |
| MIMAT0000858 | rno-miR-181a-5p | 171115 | Pde5a |
| MIMAT0000858 | rno-miR-181a-5p | 362687 | Sfrs7 |
| MIMAT0000858 | rno-miR-181a-5p | 25026 | Adm |
| MIMAT0000858 | rno-miR-181a-5p | 393087 | Pcdha5 |
| MIMAT0000858 | rno-miR-181a-5p | 64527 | Pdap1 |
| MIMAT0000858 | rno-miR-181a-5p | 361888 | Sfrs12ip1 |
| MIMAT0000858 | rno-miR-181a-5p | 56010 | Ywhag |
| MIMAT0000858 | rno-miR-181a-5p | 64053 | Dlg2 |
| MIMAT0000858 | rno-miR-181a-5p | 24513 | Ivd |
| MIMAT0000858 | rno-miR-181a-5p | 314352 | Sel1l |
| MIMAT0000858 | rno-miR-181a-5p | 298012 | Rad23b |
| MIMAT0000858 | rno-miR-181a-5p | 305131 | Lrrc8d |
| MIMAT0000858 | rno-miR-181a-5p | 499563 | Zfp458 |
| MIMAT0000858 | rno-miR-181a-5p | 29733 | S1pr1 |
| MIMAT0000858 | rno-miR-181a-5p | 64124 | Eltd1 |
| MIMAT0000858 | rno-miR-181a-5p | 363869 | Ubl3 |
| MIMAT0000858 | rno-miR-181a-5p | 293067 | Zfand6 |
| MIMAT0000858 | rno-miR-181a-5p | 364137 | Tmem165 |
| MIMAT0000858 | rno-miR-181a-5p | 290985 | Isca1 |
| MIMAT0000858 | rno-miR-181a-5p | 312824 | Recql |
| MIMAT0000858 | rno-miR-181a-5p | 500280 | Lrrn1 |
| MIMAT0000858 | rno-miR-181a-5p | 311437 | Rassf2 |
| MIMAT0000858 | rno-miR-181a-5p | 286973 | Elavl2 |
| MIMAT0000858 | rno-miR-181a-5p | 117050 | Arl5a |
| MIMAT0000858 | rno-miR-181a-5p | 170906 | Zdhhc7 |
| MIMAT0000858 | rno-miR-181a-5p | 500826 | Galnt4 |
| MIMAT0000858 | rno-miR-181a-5p | 83476 | Cyr61 |
| MIMAT0000858 | rno-miR-181a-5p | 81655 | Dync1li2 |
| MIMAT0000858 | rno-miR-181a-5p | 29748 | Ppp3r1 |
| MIMAT0000858 | rno-miR-181a-5p | 60331 | Atxn3 |
| MIMAT0000858 | rno-miR-181a-5p | 24404 | Gpx1 |
| MIMAT0000858 | rno-miR-181a-5p | 79242 | Hpgd |
| MIMAT0000858 | rno-miR-181a-5p | 393089 | Pcdha7 |
| MIMAT0000858 | rno-miR-181a-5p | 393091 | Pcdhac1 |
| MIMAT0000858 | rno-miR-181a-5p | 64536 | Esm1 |
| MIMAT0000858 | rno-miR-181a-5p | 286939 | Asam |
| MIMAT0000858 | rno-miR-181a-5p | 85260 | Entpd6 |
| MIMAT0000858 | rno-miR-181a-5p | 81830 | Rab11a |
| MIMAT0000858 | rno-miR-181a-5p | 170816 | Olr59 |
| MIMAT0000858 | rno-miR-181a-5p | 246254 | Gpsm1 |
| MIMAT0000858 | rno-miR-181a-5p | 361301 | RGD1310571 |
| MIMAT0000858 | rno-miR-181a-5p | 293343 | Fam160a2 |
| MIMAT0000858 | rno-miR-181a-5p | 362962 | Cbx7 |
| MIMAT0000858 | rno-miR-181a-5p | 282845 | Rnf34 |
| MIMAT0000858 | rno-miR-181a-5p | 363455 | Chrdl1 |
| MIMAT0000858 | rno-miR-181a-5p | 64553 | Akap6 |
| MIMAT0000858 | rno-miR-181a-5p | 310378 | Nnt |
| MIMAT0000858 | rno-miR-181a-5p | 116658 | Cntn4 |
| MIMAT0000858 | rno-miR-181a-5p | 192361 | Ppp1r2 |
| MIMAT0000858 | rno-miR-181a-5p | 140734 | Dynll2 |
| MIMAT0000858 | rno-miR-181a-5p | 117041 | Nln |
| MIMAT0000858 | rno-miR-181a-5p | 291946 | Tmem184c |
| MIMAT0000858 | rno-miR-181a-5p | 25647 | Il7 |
| MIMAT0000858 | rno-miR-181a-5p | 25428 | Cyp7a1 |
| MIMAT0000858 | rno-miR-181a-5p | 156435 | Tmprss2 |
| MIMAT0000858 | rno-miR-181a-5p | 25152 | Map1a |
| MIMAT0000858 | rno-miR-181a-5p | 360764 | Znf655 |
| MIMAT0000858 | rno-miR-181a-5p | 116742 | Pcdha13 |
| MIMAT0000858 | rno-miR-181a-5p | 24479 | Idh1 |
| MIMAT0000858 | rno-miR-181a-5p | 58853 | Nr4a3 |
| MIMAT0000858 | rno-miR-181a-5p | 65272 | Kcnk10 |
| MIMAT0000858 | rno-miR-181a-5p | 25288 | Acsl1 |
| MIMAT0000858 | rno-miR-181a-5p | 362453 | Crebl2 |
| MIMAT0000858 | rno-miR-181a-5p | 363269 | Sp100 |
| MIMAT0000858 | rno-miR-181a-5p | 303831 | Eif4a2 |
| MIMAT0000862 | rno-miR-185-5p | 24334 | Eno2 |
| MIMAT0000862 | rno-miR-185-5p | 83589 | Apba1 |
| MIMAT0000862 | rno-miR-185-5p | 393092 | Pcdhac2 |
| MIMAT0000862 | rno-miR-185-5p | 29582 | Mgat3 |
| MIMAT0000862 | rno-miR-185-5p | 252917 | Nr1d1 |
| MIMAT0000862 | rno-miR-185-5p | 56611 | Anxa2 |
| MIMAT0000862 | rno-miR-185-5p | 116778 | Pcdha10 |
| MIMAT0000862 | rno-miR-185-5p | 365493 | RGD1308127 |
| MIMAT0000862 | rno-miR-185-5p | 361845 | Eif4ebp2 |
| MIMAT0000862 | rno-miR-185-5p | 246142 | Bmf |
| MIMAT0000862 | rno-miR-185-5p | 500925 | MGC114464 |
| MIMAT0000862 | rno-miR-185-5p | 24213 | Atp1a3 |
| MIMAT0000862 | rno-miR-185-5p | 362326 | Tspan12 |
| MIMAT0000862 | rno-miR-185-5p | 313647 | Hp1bp3 |
| MIMAT0000862 | rno-miR-185-5p | 300666 | C2cd2l |
| MIMAT0000862 | rno-miR-185-5p | 29619 | Btg2 |
| MIMAT0000862 | rno-miR-185-5p | 393086 | Pcdha2 |
| MIMAT0000862 | rno-miR-185-5p | 29423 | Gap43 |
| MIMAT0000862 | rno-miR-185-5p | 171018 | Zfp384 |
| MIMAT0000862 | rno-miR-185-5p | 314641 | Pip5k1c |
| MIMAT0000862 | rno-miR-185-5p | 81515 | Lyn |
| MIMAT0000862 | rno-miR-185-5p | 24499 | Il6ra |
| MIMAT0000862 | rno-miR-185-5p | 353229 | Sgms1 |
| MIMAT0000862 | rno-miR-185-5p | 291180 | Epdr1 |
| MIMAT0000862 | rno-miR-185-5p | 393085 | Pcdha1 |
| MIMAT0000862 | rno-miR-185-5p | 116741 | Pcdha4 |
| MIMAT0000862 | rno-miR-185-5p | 140665 | Rab3d |
| MIMAT0000862 | rno-miR-185-5p | 83764 | Flot2 |
| MIMAT0000862 | rno-miR-185-5p | 84354 | Ssbp3 |
| MIMAT0000862 | rno-miR-185-5p | 24311 | Ddc |
| MIMAT0000862 | rno-miR-185-5p | 117855 | Sf1 |
| MIMAT0000862 | rno-miR-185-5p | 24923 | Stx1b |
| MIMAT0000862 | rno-miR-185-5p | 116779 | Pcdha12 |
| MIMAT0000862 | rno-miR-185-5p | 312711 | RGD1304952 |
| MIMAT0000862 | rno-miR-185-5p | 313672 | LOC313672 |
| MIMAT0000862 | rno-miR-185-5p | 116780 | Pcdha3 |
| MIMAT0000862 | rno-miR-185-5p | 313264 | Trim32 |
| MIMAT0000862 | rno-miR-185-5p | 84396 | Atp1b4 |
| MIMAT0000862 | rno-miR-185-5p | 365751 | Trim55 |
| MIMAT0000862 | rno-miR-185-5p | 361992 | Them4 |
| MIMAT0000862 | rno-miR-185-5p | 361765 | Btrc |
| MIMAT0000862 | rno-miR-185-5p | 292686 | Fbxo46 |
| MIMAT0000862 | rno-miR-185-5p | 305434 | Jakmip1 |
| MIMAT0000862 | rno-miR-185-5p | 307652 | Nip30 |
| MIMAT0000862 | rno-miR-185-5p | 79215 | Mtpn |
| MIMAT0000862 | rno-miR-185-5p | 366854 | Fbxo7 |
| MIMAT0000862 | rno-miR-185-5p | 311670 | Cstf1 |
| MIMAT0000862 | rno-miR-185-5p | 362048 | Tspan5 |
| MIMAT0000862 | rno-miR-185-5p | 79220 | Grid2 |
| MIMAT0000862 | rno-miR-185-5p | 494320 | Ccdc8 |
| MIMAT0000862 | rno-miR-185-5p | 64364 | Pllp |
| MIMAT0000862 | rno-miR-185-5p | 316077 | Entpd3 |
| MIMAT0000862 | rno-miR-185-5p | 362957 | Ankrd54 |
| MIMAT0000862 | rno-miR-185-5p | 29511 | Padi2 |
| MIMAT0000862 | rno-miR-185-5p | 393088 | Pcdha6 |
| MIMAT0000862 | rno-miR-185-5p | 83801 | Ptms |
| MIMAT0000862 | rno-miR-185-5p | 362434 | Lpcat3 |
| MIMAT0000862 | rno-miR-185-5p | 29175 | Ctsk |
| MIMAT0000862 | rno-miR-185-5p | 116676 | Aldh1a2 |
| MIMAT0000862 | rno-miR-185-5p | 63837 | Tagln3 |
| MIMAT0000862 | rno-miR-185-5p | 362281 | Rae1 |
| MIMAT0000862 | rno-miR-185-5p | 64549 | Inhbc |
| MIMAT0000862 | rno-miR-185-5p | 113970 | Magi2 |
| MIMAT0000862 | rno-miR-185-5p | 54297 | Rgs8 |
| MIMAT0000862 | rno-miR-185-5p | 369016 | Myadm |
| MIMAT0000862 | rno-miR-185-5p | 293950 | Morn4 |
| MIMAT0000862 | rno-miR-185-5p | 83502 | Cdh1 |
| MIMAT0000862 | rno-miR-185-5p | 56806 | Adam2 |
| MIMAT0000862 | rno-miR-185-5p | 170566 | Slc16a10 |
| MIMAT0000862 | rno-miR-185-5p | 294679 | Ankra2 |
| MIMAT0000862 | rno-miR-185-5p | 56064 | Nptn |
| MIMAT0000862 | rno-miR-185-5p | 63996 | Smc1a |
| MIMAT0000862 | rno-miR-185-5p | 64154 | Gosr2 |
| MIMAT0000862 | rno-miR-185-5p | 25181 | Bgn |
| MIMAT0000862 | rno-miR-185-5p | 64471 | Plekhb1 |
| MIMAT0000862 | rno-miR-185-5p | 300078 | Dnal4 |
| MIMAT0000862 | rno-miR-185-5p | 366568 | Slc30a3 |
| MIMAT0000862 | rno-miR-185-5p | 305302 | Rasl11b |
| MIMAT0000862 | rno-miR-185-5p | 307491 | Hars2l |
| MIMAT0000862 | rno-miR-185-5p | 64203 | Bcat2 |
| MIMAT0000862 | rno-miR-185-5p | 85256 | Slc2a8 |
| MIMAT0000862 | rno-miR-185-5p | 393087 | Pcdha5 |
| MIMAT0000862 | rno-miR-185-5p | 50563 | Gja5 |
| MIMAT0000862 | rno-miR-185-5p | 24694 | Pth |
| MIMAT0000862 | rno-miR-185-5p | 360750 | Ccl25 |
| MIMAT0000862 | rno-miR-185-5p | 300674 | Arcn1 |
| MIMAT0000862 | rno-miR-185-5p | 299201 | Dlst |
| MIMAT0000862 | rno-miR-185-5p | 29525 | Pitpna |
| MIMAT0000862 | rno-miR-185-5p | 360800 | Sumf2 |
| MIMAT0000862 | rno-miR-185-5p | 64356 | Nrgn |
| MIMAT0000862 | rno-miR-185-5p | 288599 | Eif4h |
| MIMAT0000862 | rno-miR-185-5p | 305889 | Thtpa |
| MIMAT0000862 | rno-miR-185-5p | 25054 | Ntrk2 |
| MIMAT0000862 | rno-miR-185-5p | 246760 | Mafk |
| MIMAT0000862 | rno-miR-185-5p | 297893 | Hdac1 |
| MIMAT0000862 | rno-miR-185-5p | 59317 | Epb4.1l1 |
| MIMAT0000862 | rno-miR-185-5p | 64134 | Xylt2 |
| MIMAT0000862 | rno-miR-185-5p | 24410 | Grin2b |
| MIMAT0000862 | rno-miR-185-5p | 366734 | Bag5 |
| MIMAT0000862 | rno-miR-185-5p | 25155 | Syk |
| MIMAT0000862 | rno-miR-185-5p | 64043 | Gcnt1 |
| MIMAT0000862 | rno-miR-185-5p | 116664 | Atp6v1f |
| MIMAT0000862 | rno-miR-185-5p | 301245 | Yipf3 |
| MIMAT0000862 | rno-miR-185-5p | 311437 | Rassf2 |
| MIMAT0000862 | rno-miR-185-5p | 497959 | Proca1 |
| MIMAT0000862 | rno-miR-185-5p | 25151 | Igf2r |
| MIMAT0000862 | rno-miR-185-5p | 24918 | Stat5a |
| MIMAT0000862 | rno-miR-185-5p | 365778 | LOC365778 |
| MIMAT0000862 | rno-miR-185-5p | 29201 | Ctf1 |
| MIMAT0000862 | rno-miR-185-5p | 298584 | Capzb |
| MIMAT0000862 | rno-miR-185-5p | 266711 | Lzts1 |
| MIMAT0000862 | rno-miR-185-5p | 297801 | Xkr4 |
| MIMAT0000862 | rno-miR-185-5p | 83627 | Galnt5 |
| MIMAT0000862 | rno-miR-185-5p | 310663 | Tnfaip8l2 |
| MIMAT0000862 | rno-miR-185-5p | 298577 | Ubxd3 |
| MIMAT0000862 | rno-miR-185-5p | 25293 | Aqp4 |
| MIMAT0000862 | rno-miR-185-5p | 81739 | P2rx3 |
| MIMAT0000862 | rno-miR-185-5p | 192350 | Crkrs |
| MIMAT0000862 | rno-miR-185-5p | 25113 | Ddn |
| MIMAT0000862 | rno-miR-185-5p | 25337 | Nfyc |
| MIMAT0000862 | rno-miR-185-5p | 393089 | Pcdha7 |
| MIMAT0000862 | rno-miR-185-5p | 79435 | RGD69425 |
| MIMAT0000862 | rno-miR-185-5p | 246047 | Calcoco1 |
| MIMAT0000862 | rno-miR-185-5p | 361881 | Zbed3 |
| MIMAT0000862 | rno-miR-185-5p | 393091 | Pcdhac1 |
| MIMAT0000862 | rno-miR-185-5p | 29632 | Hsd3b6 |
| MIMAT0000862 | rno-miR-185-5p | 64199 | Gng11 |
| MIMAT0000862 | rno-miR-185-5p | 60562 | Stx6 |
| MIMAT0000862 | rno-miR-185-5p | 83626 | Ugcg |
| MIMAT0000862 | rno-miR-185-5p | 296161 | Ubox5 |
| MIMAT0000862 | rno-miR-185-5p | 63836 | Actn4 |
| MIMAT0000862 | rno-miR-185-5p | 304346 | Mblac1 |
| MIMAT0000862 | rno-miR-185-5p | 24681 | Prkcg |
| MIMAT0000862 | rno-miR-185-5p | 83506 | Camkk2 |
| MIMAT0000862 | rno-miR-185-5p | 64553 | Akap6 |
| MIMAT0000862 | rno-miR-185-5p | 311903 | Mrrf |
| MIMAT0000862 | rno-miR-185-5p | 29592 | Bcat1 |
| MIMAT0000862 | rno-miR-185-5p | 66013 | Arhgef9 |
| MIMAT0000862 | rno-miR-185-5p | 288700 | Rab35 |
| MIMAT0000862 | rno-miR-185-5p | 364719 | Prss16 |
| MIMAT0000862 | rno-miR-185-5p | 316067 | Xylb |
| MIMAT0000862 | rno-miR-185-5p | 29745 | Sema4f |
| MIMAT0000862 | rno-miR-185-5p | 65042 | Sfxn3 |
| MIMAT0000862 | rno-miR-185-5p | 140734 | Dynll2 |
| MIMAT0000862 | rno-miR-185-5p | 24827 | Tgfa |
| MIMAT0000862 | rno-miR-185-5p | 252924 | Nr2c1 |
| MIMAT0000862 | rno-miR-185-5p | 54276 | Neurod2 |
| MIMAT0000862 | rno-miR-185-5p | 25049 | Atxn1 |
| MIMAT0000862 | rno-miR-185-5p | 64152 | Chp |
| MIMAT0000862 | rno-miR-185-5p | 29415 | S1pr2 |
| MIMAT0000862 | rno-miR-185-5p | 311456 | Mkks |
| MIMAT0000862 | rno-miR-185-5p | 308995 | Itgal |
| MIMAT0000862 | rno-miR-185-5p | 291703 | Ercc3 |
| MIMAT0000862 | rno-miR-185-5p | 116742 | Pcdha13 |
| MIMAT0000862 | rno-miR-185-5p | 25718 | Igf1r |
| MIMAT0000862 | rno-miR-185-5p | 289883 | Tmed5 |
| MIMAT0000862 | rno-miR-185-5p | 85248 | Kif3c |
| MIMAT0000862 | rno-miR-185-5p | 29245 | Klk6 |
| MIMAT0000862 | rno-miR-185-5p | 81519 | Mgat1 |
| MIMAT0000862 | rno-miR-185-5p | 117556 | Sv2b |
| MIMAT0000862 | rno-miR-185-5p | 192280 | Ppp1r3b |
| MIMAT0000862 | rno-miR-185-5p | 292878 | MGC93975 |
| MIMAT0000862 | rno-miR-185-5p | 85428 | Rhoq |
| MIMAT0000862 | rno-miR-185-5p | 292728 | Cyp2b21 |
| MIMAT0000874 | rno-miR-200a-3p | 24483 | Igf2 |
| MIMAT0000874 | rno-miR-200a-3p | 171060 | Il13ra2 |
| MIMAT0000874 | rno-miR-200a-3p | 312903 | Tram1 |
| MIMAT0000874 | rno-miR-200a-3p | 117104 | Ppp2r2a |
| MIMAT0000874 | rno-miR-200a-3p | 304543 | Mlec |
| MIMAT0000874 | rno-miR-200a-3p | 84487 | Slc17a6 |
| MIMAT0000874 | rno-miR-200a-3p | 365493 | RGD1308127 |
| MIMAT0000874 | rno-miR-200a-3p | 288233 | Wrb |
| MIMAT0000874 | rno-miR-200a-3p | 171121 | Ppp6c |
| MIMAT0000874 | rno-miR-200a-3p | 298792 | Ypel5 |
| MIMAT0000874 | rno-miR-200a-3p | 25514 | Lypla1 |
| MIMAT0000874 | rno-miR-200a-3p | 497815 | Nrcam |
| MIMAT0000874 | rno-miR-200a-3p | 293098 | Tmem135 |
| MIMAT0000874 | rno-miR-200a-3p | 362750 | Atl1 |
| MIMAT0000874 | rno-miR-200a-3p | 25694 | Has2 |
| MIMAT0000874 | rno-miR-200a-3p | 29170 | Aqp6 |
| MIMAT0000874 | rno-miR-200a-3p | 360854 | Arpc5 |
| MIMAT0000874 | rno-miR-200a-3p | 81809 | Tgfb2 |
| MIMAT0000874 | rno-miR-200a-3p | 299857 | Shmt2 |
| MIMAT0000874 | rno-miR-200a-3p | 83580 | Thbd |
| MIMAT0000874 | rno-miR-200a-3p | 296731 | Nub1 |
| MIMAT0000874 | rno-miR-200a-3p | 114561 | Pitpnb |
| MIMAT0000874 | rno-miR-200a-3p | 363545 | Nat15 |
| MIMAT0000874 | rno-miR-200a-3p | 24516 | Jun |
| MIMAT0000874 | rno-miR-200a-3p | 170956 | Yt521 |
| MIMAT0000874 | rno-miR-200a-3p | 29637 | Hmgcs1 |
| MIMAT0000874 | rno-miR-200a-3p | 361944 | Elf2 |
| MIMAT0000874 | rno-miR-200a-3p | 83534 | Tpp1 |
| MIMAT0000874 | rno-miR-200a-3p | 498160 | Zkscan1 |
| MIMAT0000874 | rno-miR-200a-3p | 114216 | S100a3 |
| MIMAT0000874 | rno-miR-200a-3p | 306809 | Bicd2 |
| MIMAT0000874 | rno-miR-200a-3p | 84587 | Plcl1 |
| MIMAT0000874 | rno-miR-200a-3p | 24856 | Ttr |
| MIMAT0000874 | rno-miR-200a-3p | 312135 | Tmem168 |
| MIMAT0000874 | rno-miR-200a-3p | 306817 | Dek |
| MIMAT0000874 | rno-miR-200a-3p | 65190 | Rsad2 |
| MIMAT0000874 | rno-miR-200a-3p | 291078 | Prpf4b |
| MIMAT0000874 | rno-miR-200a-3p | 29463 | Ptp4a1 |
| MIMAT0000874 | rno-miR-200a-3p | 114021 | Ebna1bp2 |
| MIMAT0000874 | rno-miR-200a-3p | 114514 | Clasp2 |
| MIMAT0000874 | rno-miR-200a-3p | 79433 | Myh10 |
| MIMAT0000874 | rno-miR-200a-3p | 29345 | Serpinh1 |
| MIMAT0000874 | rno-miR-200a-3p | 170704 | Hrh4 |
| MIMAT0000874 | rno-miR-200a-3p | 24672 | Ppp2ca |
| MIMAT0000874 | rno-miR-200a-3p | 116506 | Calcr |
| MIMAT0000874 | rno-miR-200a-3p | 116509 | Slc6a9 |
| MIMAT0000874 | rno-miR-200a-3p | 26759 | Acot7 |
| MIMAT0000874 | rno-miR-200a-3p | 260323 | Snx27 |
| MIMAT0000874 | rno-miR-200a-3p | 25389 | Atf3 |
| MIMAT0000874 | rno-miR-200a-3p | 360868 | Sft2d2 |
| MIMAT0000874 | rno-miR-200a-3p | 369016 | Myadm |
| MIMAT0000874 | rno-miR-200a-3p | 59265 | Phlpp1 |
| MIMAT0000874 | rno-miR-200a-3p | 24413 | Nr3c1 |
| MIMAT0000874 | rno-miR-200a-3p | 140670 | Ap2b1 |
| MIMAT0000874 | rno-miR-200a-3p | 245959 | Slc25a3 |
| MIMAT0000874 | rno-miR-200a-3p | 65137 | Ruvbl1 |
| MIMAT0000874 | rno-miR-200a-3p | 191575 | Nme1 |
| MIMAT0000874 | rno-miR-200a-3p | 29564 | Dync1i1 |
| MIMAT0000874 | rno-miR-200a-3p | 24188 | Aldh1a1 |
| MIMAT0000874 | rno-miR-200a-3p | 361810 | Fkbp5 |
| MIMAT0000874 | rno-miR-200a-3p | 311346 | Lrrc57 |
| MIMAT0000874 | rno-miR-200a-3p | 311846 | Lrrc8a |
| MIMAT0000874 | rno-miR-200a-3p | 93667 | Olfm1 |
| MIMAT0000874 | rno-miR-200a-3p | 83842 | Crot |
| MIMAT0000874 | rno-miR-200a-3p | 24942 | Chm |
| MIMAT0000874 | rno-miR-200a-3p | 291948 | Pgrmc1 |
| MIMAT0000874 | rno-miR-200a-3p | 29735 | Slc16a7 |
| MIMAT0000874 | rno-miR-200a-3p | 315608 | Ube4a |
| MIMAT0000874 | rno-miR-200a-3p | 313588 | Snip1 |
| MIMAT0000874 | rno-miR-200a-3p | 56010 | Ywhag |
| MIMAT0000874 | rno-miR-200a-3p | 25054 | Ntrk2 |
| MIMAT0000874 | rno-miR-200a-3p | 171458 | Ipmk |
| MIMAT0000874 | rno-miR-200a-3p | 296478 | Zgpat |
| MIMAT0000874 | rno-miR-200a-3p | 302669 | Car5b |
| MIMAT0000874 | rno-miR-200a-3p | 25187 | Htr2c |
| MIMAT0000874 | rno-miR-200a-3p | 117596 | Atp6v1b2 |
| MIMAT0000874 | rno-miR-200a-3p | 300886 | Mthfs |
| MIMAT0000874 | rno-miR-200a-3p | 500941 | MGC105560 |
| MIMAT0000874 | rno-miR-200a-3p | 499602 | LOC499602 |
| MIMAT0000874 | rno-miR-200a-3p | 29149 | Strn |
| MIMAT0000874 | rno-miR-200a-3p | 117559 | Sv2a |
| MIMAT0000874 | rno-miR-200a-3p | 362061 | Cryz |
| MIMAT0000874 | rno-miR-200a-3p | 252916 | Rab38 |
| MIMAT0000874 | rno-miR-200a-3p | 296315 | RGD1307752 |
| MIMAT0000874 | rno-miR-200a-3p | 286973 | Elavl2 |
| MIMAT0000874 | rno-miR-200a-3p | 24918 | Stat5a |
| MIMAT0000874 | rno-miR-200a-3p | 64551 | Sept7 |
| MIMAT0000874 | rno-miR-200a-3p | 312495 | Cyp26b1 |
| MIMAT0000874 | rno-miR-200a-3p | 170906 | Zdhhc7 |
| MIMAT0000874 | rno-miR-200a-3p | 54398 | Ppt2 |
| MIMAT0000874 | rno-miR-200a-3p | 307505 | Ctnna1 |
| MIMAT0000874 | rno-miR-200a-3p | 287765 | Ddx5 |
| MIMAT0000874 | rno-miR-200a-3p | 246334 | Tp63 |
| MIMAT0000874 | rno-miR-200a-3p | 25615 | Sdc2 |
| MIMAT0000874 | rno-miR-200a-3p | 315741 | Paqr5 |
| MIMAT0000874 | rno-miR-200a-3p | 54284 | Pitx2 |
| MIMAT0000874 | rno-miR-200a-3p | 64200 | Hnrnpf |
| MIMAT0000874 | rno-miR-200a-3p | 291534 | Rnmt |
| MIMAT0000874 | rno-miR-200a-3p | 116482 | Sacm1l |
| MIMAT0000874 | rno-miR-200a-3p | 298943 | Bcap29 |
| MIMAT0000874 | rno-miR-200a-3p | 306720 | Ctsm |
| MIMAT0000874 | rno-miR-200a-3p | 140941 | Siah1a |
| MIMAT0000874 | rno-miR-200a-3p | 65196 | B4galt6 |
| MIMAT0000874 | rno-miR-200a-3p | 81684 | Mipep |
| MIMAT0000874 | rno-miR-200a-3p | 116744 | Lpar1 |
| MIMAT0000874 | rno-miR-200a-3p | 29544 | Tspyl1 |
| MIMAT0000874 | rno-miR-200a-3p | 116551 | Pdk1 |
| MIMAT0000874 | rno-miR-200a-3p | 60443 | Epn2 |
| MIMAT0000874 | rno-miR-200a-3p | 60562 | Stx6 |
| MIMAT0000874 | rno-miR-200a-3p | 317385 | Fus |
| MIMAT0000874 | rno-miR-200a-3p | 290032 | Fam12b |
| MIMAT0000874 | rno-miR-200a-3p | 297123 | Fkbp9 |
| MIMAT0000874 | rno-miR-200a-3p | 64469 | Slc30a4 |
| MIMAT0000874 | rno-miR-200a-3p | 29592 | Bcat1 |
| MIMAT0000874 | rno-miR-200a-3p | 83685 | Capn6 |
| MIMAT0000874 | rno-miR-200a-3p | 25558 | Stxbp1 |
| MIMAT0000874 | rno-miR-200a-3p | 50655 | Aco1 |
| MIMAT0000874 | rno-miR-200a-3p | 58976 | Slc30a1 |
| MIMAT0000874 | rno-miR-200a-3p | 304791 | Ripk5 |
| MIMAT0000874 | rno-miR-200a-3p | 117514 | Txnip |
| MIMAT0000874 | rno-miR-200a-3p | 289881 | Dr1 |
| MIMAT0000874 | rno-miR-200a-3p | 64152 | Chp |
| MIMAT0000874 | rno-miR-200a-3p | 84352 | Col1a2 |
| MIMAT0000874 | rno-miR-200a-3p | 60586 | Clcn4-2 |
| MIMAT0000874 | rno-miR-200a-3p | 29657 | Arntl |
| MIMAT0000874 | rno-miR-200a-3p | 171517 | Gpc2 |
| MIMAT0000874 | rno-miR-200a-3p | 66025 | Lpar3 |
| MIMAT0000874 | rno-miR-200a-3p | 79119 | Arf2 |
| MIMAT0000874 | rno-miR-200a-3p | 289883 | Tmed5 |
| MIMAT0000874 | rno-miR-200a-3p | 24184 | Ak2 |
| MIMAT0000874 | rno-miR-200a-3p | 361110 | Tmem110 |
| MIMAT0000874 | rno-miR-200a-3p | 361442 | Sipa1l2 |
| MIMAT0000874 | rno-miR-200a-3p | 497757 | Gucy1a3 |
| MIMAT0000874 | rno-miR-200a-3p | 24831 | Thrb |
| MIMAT0000874 | rno-miR-200a-3p | 25110 | Klrd1 |
| MIMAT0000874 | rno-miR-200a-3p | 498564 | Itgbl1 |
| MIMAT0000874 | rno-miR-200a-3p | 116668 | Myt1l |
| MIMAT0000895 | rno-miR-291a-3p | 363035 | Zbtb44 |
| MIMAT0000895 | rno-miR-291a-3p | 393092 | Pcdhac2 |
| MIMAT0000895 | rno-miR-291a-3p | 308023 | Ssx2ip |
| MIMAT0000895 | rno-miR-291a-3p | 298934 | Adi1 |
| MIMAT0000895 | rno-miR-291a-3p | 116778 | Pcdha10 |
| MIMAT0000895 | rno-miR-291a-3p | 298894 | Mycn |
| MIMAT0000895 | rno-miR-291a-3p | 312694 | Necap1 |
| MIMAT0000895 | rno-miR-291a-3p | 361238 | RGD1311307 |
| MIMAT0000895 | rno-miR-291a-3p | 83526 | Atrn |
| MIMAT0000895 | rno-miR-291a-3p | 170820 | Prdm4 |
| MIMAT0000895 | rno-miR-291a-3p | 362891 | Os9 |
| MIMAT0000895 | rno-miR-291a-3p | 81825 | Cirbp |
| MIMAT0000895 | rno-miR-291a-3p | 50659 | Nr2c2 |
| MIMAT0000895 | rno-miR-291a-3p | 29618 | Btg1 |
| MIMAT0000895 | rno-miR-291a-3p | 171121 | Ppp6c |
| MIMAT0000895 | rno-miR-291a-3p | 361233 | Ssr1 |
| MIMAT0000895 | rno-miR-291a-3p | 29458 | Neurod1 |
| MIMAT0000895 | rno-miR-291a-3p | 393086 | Pcdha2 |
| MIMAT0000895 | rno-miR-291a-3p | 50658 | Mapk9 |
| MIMAT0000895 | rno-miR-291a-3p | 298317 | Ube2a |
| MIMAT0000895 | rno-miR-291a-3p | 83712 | Rbbp7 |
| MIMAT0000895 | rno-miR-291a-3p | 393085 | Pcdha1 |
| MIMAT0000895 | rno-miR-291a-3p | 192357 | Gmpr2 |
| MIMAT0000895 | rno-miR-291a-3p | 116741 | Pcdha4 |
| MIMAT0000895 | rno-miR-291a-3p | 85264 | Abcg1 |
| MIMAT0000895 | rno-miR-291a-3p | 308937 | Wee1 |
| MIMAT0000895 | rno-miR-291a-3p | 287276 | Sar1b |
| MIMAT0000895 | rno-miR-291a-3p | 294074 | Cep55 |
| MIMAT0000895 | rno-miR-291a-3p | 498160 | Zkscan1 |
| MIMAT0000895 | rno-miR-291a-3p | 64476 | Mfn2 |
| MIMAT0000895 | rno-miR-291a-3p | 116779 | Pcdha12 |
| MIMAT0000895 | rno-miR-291a-3p | 362521 | Tmem38b |
| MIMAT0000895 | rno-miR-291a-3p | 116780 | Pcdha3 |
| MIMAT0000895 | rno-miR-291a-3p | 311872 | Zbtb43 |
| MIMAT0000895 | rno-miR-291a-3p | 81816 | Ube2b |
| MIMAT0000895 | rno-miR-291a-3p | 24316 | Drd1a |
| MIMAT0000895 | rno-miR-291a-3p | 303346 | Ccdc55 |
| MIMAT0000895 | rno-miR-291a-3p | 24874 | Vhl |
| MIMAT0000895 | rno-miR-291a-3p | 83533 | Aifm1 |
| MIMAT0000895 | rno-miR-291a-3p | 315265 | Twf1 |
| MIMAT0000895 | rno-miR-291a-3p | 362957 | Ankrd54 |
| MIMAT0000895 | rno-miR-291a-3p | 393088 | Pcdha6 |
| MIMAT0000895 | rno-miR-291a-3p | 286994 | Lgr4 |
| MIMAT0000895 | rno-miR-291a-3p | 315134 | Josd1 |
| MIMAT0000895 | rno-miR-291a-3p | 116509 | Slc6a9 |
| MIMAT0000895 | rno-miR-291a-3p | 305482 | Mtmr3 |
| MIMAT0000895 | rno-miR-291a-3p | 362129 | Gtdc1 |
| MIMAT0000895 | rno-miR-291a-3p | 85245 | Kpna2 |
| MIMAT0000895 | rno-miR-291a-3p | 25139 | Slc2a4 |
| MIMAT0000895 | rno-miR-291a-3p | 25584 | F3 |
| MIMAT0000895 | rno-miR-291a-3p | 116500 | Snap29 |
| MIMAT0000895 | rno-miR-291a-3p | 444983 | Apold1 |
| MIMAT0000895 | rno-miR-291a-3p | 83842 | Crot |
| MIMAT0000895 | rno-miR-291a-3p | 303902 | Dirc2 |
| MIMAT0000895 | rno-miR-291a-3p | 57027 | Adam17 |
| MIMAT0000895 | rno-miR-291a-3p | 316153 | Sult1c2a |
| MIMAT0000895 | rno-miR-291a-3p | 393087 | Pcdha5 |
| MIMAT0000895 | rno-miR-291a-3p | 365963 | Lhx8 |
| MIMAT0000895 | rno-miR-291a-3p | 29748 | Ppp3r1 |
| MIMAT0000895 | rno-miR-291a-3p | 298012 | Rad23b |
| MIMAT0000895 | rno-miR-291a-3p | 315159 | Tob2 |
| MIMAT0000895 | rno-miR-291a-3p | 25216 | Sdc1 |
| MIMAT0000895 | rno-miR-291a-3p | 171070 | Ptpn21 |
| MIMAT0000895 | rno-miR-291a-3p | 362061 | Cryz |
| MIMAT0000895 | rno-miR-291a-3p | 252920 | Olfm3 |
| MIMAT0000895 | rno-miR-291a-3p | 286973 | Elavl2 |
| MIMAT0000895 | rno-miR-291a-3p | 114246 | Trpv6 |
| MIMAT0000895 | rno-miR-291a-3p | 315939 | Acpl2 |
| MIMAT0000895 | rno-miR-291a-3p | 312495 | Cyp26b1 |
| MIMAT0000895 | rno-miR-291a-3p | 359726 | Rnasel |
| MIMAT0000895 | rno-miR-291a-3p | 306695 | Zfp367 |
| MIMAT0000895 | rno-miR-291a-3p | 84481 | Arid4b |
| MIMAT0000895 | rno-miR-291a-3p | 299618 | Mknk2 |
| MIMAT0000895 | rno-miR-291a-3p | 393089 | Pcdha7 |
| MIMAT0000895 | rno-miR-291a-3p | 393091 | Pcdhac1 |
| MIMAT0000895 | rno-miR-291a-3p | 81830 | Rab11a |
| MIMAT0000895 | rno-miR-291a-3p | 288182 | Cldnd1 |
| MIMAT0000895 | rno-miR-291a-3p | 307614 | Cdh16 |
| MIMAT0000895 | rno-miR-291a-3p | 296616 | Cercam |
| MIMAT0000895 | rno-miR-291a-3p | 303567 | Tmub2 |
| MIMAT0000895 | rno-miR-291a-3p | 499331 | LOC499331 |
| MIMAT0000895 | rno-miR-291a-3p | 25565 | Tle4 |
| MIMAT0000895 | rno-miR-291a-3p | 81758 | Rbl2 |
| MIMAT0000895 | rno-miR-291a-3p | 29480 | Rgs4 |
| MIMAT0000895 | rno-miR-291a-3p | 85426 | Slc5a7 |
| MIMAT0000895 | rno-miR-291a-3p | 117514 | Txnip |
| MIMAT0000895 | rno-miR-291a-3p | 89843 | Cxadr |
| MIMAT0000895 | rno-miR-291a-3p | 294289 | Zbtb9 |
| MIMAT0000895 | rno-miR-291a-3p | 309196 | Ttc9c |
| MIMAT0000895 | rno-miR-291a-3p | 287828 | Hn1 |
| MIMAT0000895 | rno-miR-291a-3p | 287543 | Tnfaip1 |
| MIMAT0000895 | rno-miR-291a-3p | 291733 | Slc39a6 |
| MIMAT0000895 | rno-miR-291a-3p | 360549 | Plscr3 |
| MIMAT0000895 | rno-miR-291a-3p | 25405 | Ccng1 |
| MIMAT0000895 | rno-miR-291a-3p | 116742 | Pcdha13 |
| MIMAT0000895 | rno-miR-291a-3p | 498999 | Ahrr |
| MIMAT0000895 | rno-miR-291a-3p | 266685 | Ugt2b5 |
| MIMAT0000895 | rno-miR-291a-3p | 314323 | Flvcr2 |
| MIMAT0000895 | rno-miR-291a-3p | 548326 | Faim3 |
| MIMAT0000895 | rno-miR-291a-3p | 58853 | Nr4a3 |
| MIMAT0000895 | rno-miR-291a-3p | 311406 | Dusp2 |
| MIMAT0000895 | rno-miR-291a-3p | 297804 | Plag1 |
| MIMAT0000895 | rno-miR-291a-3p | 301674 | Fbxo11 |
| MIMAT0000895 | rno-miR-291a-3p | 54190 | Rabep1 |
| MIMAT0000895 | rno-miR-291a-3p | 84026 | Dnaja2 |
| MIMAT0000895 | rno-miR-291a-3p | 305816 | Ddhd1 |
| MIMAT0000895 | rno-miR-291a-3p | 305861 | Mudeng |
| MIMAT0000895 | rno-miR-291a-3p | 362418 | Irak2 |
| MIMAT0000895 | rno-miR-291a-3p | 298098 | Pole3 |
| MIMAT0004742 | rno-miR-296-3p | 114637 | Hmgn2 |
| MIMAT0004742 | rno-miR-296-3p | 360985 | Tmem17 |
| MIMAT0004742 | rno-miR-296-3p | 361120 | Eps15l1 |
| MIMAT0004742 | rno-miR-296-3p | 89842 | Mbtps1 |
| MIMAT0004742 | rno-miR-296-3p | 317218 | Itm2a |
| MIMAT0004742 | rno-miR-296-3p | 27137 | Eif2ak1 |
| MIMAT0004742 | rno-miR-296-3p | 29340 | Prkce |
| MIMAT0004742 | rno-miR-296-3p | 116589 | Serpinb5 |
| MIMAT0004742 | rno-miR-296-3p | 64076 | Slc26a1 |
| MIMAT0004742 | rno-miR-296-3p | 298851 | RGD1309228 |
| MIMAT0004742 | rno-miR-296-3p | 315134 | Josd1 |
| MIMAT0004742 | rno-miR-296-3p | 64507 | Fmod |
| MIMAT0004742 | rno-miR-296-3p | 502970 | Angptl3 |
| MIMAT0004742 | rno-miR-296-3p | 360750 | Ccl25 |
| MIMAT0004742 | rno-miR-296-3p | 499300 | Ptprcap |
| MIMAT0004742 | rno-miR-296-3p | 310721 | LOC310721 |
| MIMAT0004742 | rno-miR-296-3p | 299201 | Dlst |
| MIMAT0004742 | rno-miR-296-3p | 25104 | Pc |
| MIMAT0004742 | rno-miR-296-3p | 140923 | Bnip3l |
| MIMAT0004742 | rno-miR-296-3p | 83500 | Slc22a8 |
| MIMAT0004742 | rno-miR-296-3p | 26989 | Cadps |
| MIMAT0004742 | rno-miR-296-3p | 114520 | Strn3 |
| MIMAT0004742 | rno-miR-296-3p | 313436 | Zcchc12 |
| MIMAT0004742 | rno-miR-296-3p | 295051 | Alg5 |
| MIMAT0004742 | rno-miR-296-3p | 297387 | Mobkl1b |
| MIMAT0004742 | rno-miR-296-3p | 81737 | Ntf3 |
| MIMAT0005303 | rno-miR-196c-5p | 362326 | Tspan12 |
| MIMAT0005303 | rno-miR-196c-5p | 29647 | Cask |
| MIMAT0005303 | rno-miR-196c-5p | 363476 | Yipf6 |
| MIMAT0005303 | rno-miR-196c-5p | 85264 | Abcg1 |
| MIMAT0005303 | rno-miR-196c-5p | 24242 | Calm1 |
| MIMAT0005303 | rno-miR-196c-5p | 294283 | Rgl2 |
| MIMAT0005303 | rno-miR-196c-5p | 83571 | Cdkn1b |
| MIMAT0005303 | rno-miR-196c-5p | 252963 | Il13ra1 |
| MIMAT0005303 | rno-miR-196c-5p | 25283 | Gclc |
| MIMAT0005303 | rno-miR-196c-5p | 29683 | Klrc1 |
| MIMAT0005303 | rno-miR-196c-5p | 362751 | Txndc1 |
| MIMAT0005303 | rno-miR-196c-5p | 24877 | Vsnl1 |
| MIMAT0005303 | rno-miR-196c-5p | 29737 | Kcnab1 |
| MIMAT0005303 | rno-miR-196c-5p | 63886 | Abcb9 |
| MIMAT0005303 | rno-miR-196c-5p | 619573 | Fam104a |
| MIMAT0005303 | rno-miR-196c-5p | 291259 | Zmynd11 |
| MIMAT0005303 | rno-miR-196c-5p | 64088 | Snx16 |
| MIMAT0005303 | rno-miR-196c-5p | 293113 | Tmem126a |
| MIMAT0005303 | rno-miR-196c-5p | 29434 | Rasgrp1 |
| MIMAT0005303 | rno-miR-196c-5p | 360834 | Vps4b |
| MIMAT0005303 | rno-miR-196c-5p | 306012 | Polr3d |
| MIMAT0005303 | rno-miR-196c-5p | 497985 | Hoxb7 |
| MIMAT0005303 | rno-miR-196c-5p | 619549 | Ppapdc2 |
| MIMAT0005303 | rno-miR-196c-5p | 292022 | Ddx19a |
| MIMAT0005303 | rno-miR-196c-5p | 25293 | Aqp4 |
| MIMAT0005303 | rno-miR-196c-5p | 293152 | Art2b |
| MIMAT0005303 | rno-miR-196c-5p | 303606 | Ccdc47 |
| MIMAT0005303 | rno-miR-196c-5p | 156435 | Tmprss2 |
| MIMAT0005303 | rno-miR-196c-5p | 24605 | Nras |
| MIMAT0000825 | rno-miR-106b-5p | 24766 | Scn2a |
| MIMAT0000825 | rno-miR-106b-5p | 365493 | Cacul1 |
| MIMAT0000825 | rno-miR-106b-5p | 500118 | Fam221a |
| MIMAT0000825 | rno-miR-106b-5p | 363227 | Nabp1 |
| MIMAT0000825 | rno-miR-106b-5p | 362007 | Sike1 |
| MIMAT0000825 | rno-miR-106b-5p | 192152 | Tgoln2 |
| MIMAT0000825 | rno-miR-106b-5p | 362626 | Rsrp1 |
| MIMAT0000830 | rno-miR-125b-5p | 306790 | Sema4d |
| MIMAT0000830 | rno-miR-125b-5p | 307833 | Ist1 |
| MIMAT0000830 | rno-miR-125b-5p | 300015 | Them6 |
| MIMAT0000830 | rno-miR-125b-5p | 286939 | Clmp |
| MIMAT0000830 | rno-miR-125b-5p | 362987 | Rabl2a |
| MIMAT0000830 | rno-miR-125b-5p | 497934 | Borcs6 |
| MIMAT0000830 | rno-miR-125b-5p | 192152 | Tgoln2 |
| MIMAT0000830 | rno-miR-125b-5p | 85265 | Ajuba |
| MIMAT0000830 | rno-miR-125b-5p | 304851 | Trmt1l |
| MIMAT0000830 | rno-miR-125b-5p | 311429 | Spef1 |
| MIMAT0000830 | rno-miR-125b-5p | 366227 | Apmap |
| MIMAT0000830 | rno-miR-125b-5p | 362011 | Dram2 |
| MIMAT0000830 | rno-miR-125b-5p | 308976 | Kdm8 |
| MIMAT0000830 | rno-miR-125b-5p | 362364 | Tril |
| MIMAT0000836 | rno-miR-130a-3p | 59329 | Sik1 |
| MIMAT0000836 | rno-miR-130a-3p | 282834 | Chmp3 |
| MIMAT0000836 | rno-miR-130a-3p | 295217 | Snapin |
| MIMAT0000836 | rno-miR-130a-3p | 360502 | Fam234a |
| MIMAT0000836 | rno-miR-130a-3p | 498749 | Tdp2 |
| MIMAT0000836 | rno-miR-130a-3p | 60586 | Clcn4 |
| MIMAT0000574 | rno-miR-140-3p | 140694 | Dnm1 |
| MIMAT0000846 | rno-miR-141-3p | 365493 | Cacul1 |
| MIMAT0000846 | rno-miR-141-3p | 500941 | Msantd4 |
| MIMAT0000846 | rno-miR-141-3p | 499602 | Abhd18 |
| MIMAT0000846 | rno-miR-141-3p | 170956 | Ythdc1 |
| MIMAT0000846 | rno-miR-141-3p | 363545 | Naa60 |
| MIMAT0000846 | rno-miR-141-3p | 140941 | Siah1 |
| MIMAT0000846 | rno-miR-141-3p | 290032 | Eddm3b |
| MIMAT0000846 | rno-miR-141-3p | 304791 | Dstyk |
| MIMAT0000846 | rno-miR-141-3p | 64152 | Chp1 |
| MIMAT0000846 | rno-miR-141-3p | 60586 | Clcn4 |
| MIMAT0000858 | rno-miR-181a-5p | 295401 | Plppr4 |
| MIMAT0000858 | rno-miR-181a-5p | 297109 | Mturn |
| MIMAT0000858 | rno-miR-181a-5p | 313840 | Rmdn2 |
| MIMAT0000858 | rno-miR-181a-5p | 500110 | Zfp467 |
| MIMAT0000858 | rno-miR-181a-5p | 362862 | Hsp90b1 |
| MIMAT0000858 | rno-miR-181a-5p | 286939 | Clmp |
| MIMAT0000858 | rno-miR-181a-5p | 361301 | Tpgs2 |
| MIMAT0000858 | rno-miR-181a-5p | 64124 | Adgrl4 |
| MIMAT0000858 | rno-miR-181a-5p | 360764 | Zfp655 |
| MIMAT0000858 | rno-miR-181a-5p | 362778 | Gskip |
| MIMAT0000858 | rno-miR-181a-5p | 362687 | Srsf7 |
| MIMAT0000858 | rno-miR-181a-5p | 361888 | Srek1ip1 |
| MIMAT0000862 | rno-miR-185-5p | 365493 | Cacul1 |
| MIMAT0000862 | rno-miR-185-5p | 500925 | Ccdc184 |
| MIMAT0000862 | rno-miR-185-5p | 24499 | Il6r |
| MIMAT0000862 | rno-miR-185-5p | 298577 | Ubxn10 |
| MIMAT0000862 | rno-miR-185-5p | 192350 | Cdk12 |
| MIMAT0000862 | rno-miR-185-5p | 312711 | Pianp |
| MIMAT0000862 | rno-miR-185-5p | 313672 | Kazn |
| MIMAT0000862 | rno-miR-185-5p | 79435 | Ube2d4 |
| MIMAT0000862 | rno-miR-185-5p | 307652 | Fam192a |
| MIMAT0000862 | rno-miR-185-5p | 59317 | Epb41l1 |
| MIMAT0000862 | rno-miR-185-5p | 64152 | Chp1 |
| MIMAT0000862 | rno-miR-185-5p | 307491 | Hars2 |
| MIMAT0000862 | rno-miR-185-5p | 292878 | Emc10 |
| MIMAT0005303 | rno-miR-196c-5p | 362751 | Tmx1 |
| MIMAT0005303 | rno-miR-196c-5p | 619549 | Plpp6 |
| MIMAT0000874 | rno-miR-200a-3p | 365493 | Cacul1 |
| MIMAT0000874 | rno-miR-200a-3p | 500941 | Msantd4 |
| MIMAT0000874 | rno-miR-200a-3p | 499602 | Abhd18 |
| MIMAT0000874 | rno-miR-200a-3p | 170956 | Ythdc1 |
| MIMAT0000874 | rno-miR-200a-3p | 363545 | Naa60 |
| MIMAT0000874 | rno-miR-200a-3p | 140941 | Siah1 |
| MIMAT0000874 | rno-miR-200a-3p | 290032 | Eddm3b |
| MIMAT0000874 | rno-miR-200a-3p | 304791 | Dstyk |
| MIMAT0000874 | rno-miR-200a-3p | 64152 | Chp1 |
| MIMAT0000874 | rno-miR-200a-3p | 60586 | Clcn4 |
| MIMAT0000895 | rno-miR-291a-3p | 361238 | Pxdc1 |
| MIMAT0000895 | rno-miR-291a-3p | 315939 | Pxylp1 |
| MIMAT0000895 | rno-miR-291a-3p | 303346 | Nsrp1 |
| MIMAT0000895 | rno-miR-291a-3p | 24316 | Drd1 |
| MIMAT0000895 | rno-miR-291a-3p | 266685 | Ugt2b17 |
| MIMAT0000895 | rno-miR-291a-3p | 548326 | Fcmr |
| MIMAT0000895 | rno-miR-291a-3p | 305861 | Ap5m1 |
| MIMAT0004742 | rno-miR-296-3p | 310721 | Fam46c |
| MIMAT0004742 | rno-miR-296-3p | 298851 | Slc35f6 |
| MIMAT0004742 | rno-miR-296-3p | 297387 | Mob1a |
| MIMAT0000811 | rno-miR-32-5p | 117536 | Nsmf |
| MIMAT0000811 | rno-miR-32-5p | 315548 | Srpra |
| MIMAT0000811 | rno-miR-32-5p | 362235 | Syndig1 |
| MIMAT0000811 | rno-miR-32-5p | 170946 | Marf1 |
| MIMAT0000811 | rno-miR-32-5p | 362535 | Lurap1l |
| MIMAT0000811 | rno-miR-32-5p | 500069 | Cep41 |
| MIMAT0000819 | rno-miR-98-5p | 619549 | Plpp6 |
| MIMAT0000819 | rno-miR-98-5p | 286910 | Smim3 |
| MIMAT0000819 | rno-miR-98-5p | 317630 | Bloc1s6 |
| MIMAT0000819 | rno-miR-98-5p | 498185 | Slc8b1 |
